# EgoActive: Integrated Wireless Wearable Sensors for Capturing Infant Egocentric Auditory–Visual Statistics and Autonomic Nervous System Function ‘in the Wild’

**DOI:** 10.3390/s23187930

**Published:** 2023-09-16

**Authors:** Elena Geangu, William A. P. Smith, Harry T. Mason, Astrid Priscilla Martinez-Cedillo, David Hunter, Marina I. Knight, Haipeng Liang, Maria del Carmen Garcia de Soria Bazan, Zion Tsz Ho Tse, Thomas Rowland, Dom Corpuz, Josh Hunter, Nishant Singh, Quoc C. Vuong, Mona Ragab Sayed Abdelgayed, David R. Mullineaux, Stephen Smith, Bruce R. Muller

**Affiliations:** 1 Psychology Department, University of York, York YO10 5DD, UK; priscilla.martinezcedillo@york.ac.uk (A.P.M.-C.); mari.garciadesoriabazan@york.ac.uk (M.d.C.G.d.S.B.); 2 Department of Computer Science, University of York, York YO10 5DD, UK; william.smith@york.ac.uk (W.A.P.S.); josh.hunter@york.ac.uk (J.H.); a0135563@u.nus.edu (M.R.S.A.); bruce.muller@york.ac.uk (B.R.M.); 3 School of Physics, Engineering and Technology, University of York, York YO10 5DD, UK; harry.mason@outlook.com (H.T.M.); david.hunter@york.ac.uk (D.H.); nishant.singh@york.ac.uk (N.S.); stephen.smith@york.ac.uk (S.S.); 4 Department of Mathematics, University of York, York YO10 5DD, UK; marina.knight@york.ac.uk (M.I.K.); david.mullineaux@york.ac.uk (D.R.M.); 5 School of Engineering and Materials Science, Queen Mary University of London, London E1 2AT, UK; haipeng.liang@qmul.ac.uk (H.L.); z.tse@qmul.ac.uk (Z.T.H.T.); 6 Protolabs, Halesfield 8, Telford TF7 4QN, UK; thomas.rowland@protolabs.co.uk (T.R.); dom.corpuz@protolabs.co.uk (D.C.); 7 Biosciences Institute, Newcastle University, Newcastle upon Tyne NE1 7RU, UK; quoc.vuong@newcastle.ac.uk

**Keywords:** infant, child, wearable sensors, egocentric view, head-mounted camera, ECG, body movement, naturalistic research methods, real-world big data, multimodal measures

## Abstract

There have been sustained efforts toward using naturalistic methods in developmental science to measure infant behaviors in the real world from an egocentric perspective because statistical regularities in the environment can shape and be shaped by the developing infant. However, there is no user-friendly and unobtrusive technology to densely and reliably sample life in the wild. To address this gap, we present the design, implementation and validation of the EgoActive platform, which addresses limitations of existing wearable technologies for developmental research. EgoActive records the active infants’ egocentric perspective of the world via a miniature wireless head-mounted camera concurrently with their physiological responses to this input via a lightweight, wireless ECG/acceleration sensor. We also provide software tools to facilitate data analyses. Our validation studies showed that the cameras and body sensors performed well. Families also reported that the platform was comfortable, easy to use and operate, and did not interfere with daily activities. The synchronized multimodal data from the EgoActive platform can help tease apart complex processes that are important for child development to further our understanding of areas ranging from executive function to emotion processing and social learning.

## 1. Introduction

One fundamental desideratum of developmental science is to formulate theoretical models that can explain phenomena occurring in infants’ and children’s everyday life—for instance, how children befriend other children, an infant’s utterance of the first word, and how infants and children use social information (e.g., facial expressions) in order to learn about the world around them. Beholding such a desideratum makes developmental research ecologically committed, and its ecological validity needs to be tested with reference to the natural environment. That environment has regularities, or natural statistics, such as the frequency of particular words spoken or occurrence of faces in an infant’s field of view, that can shape and be shaped by the developing infant [1]. Historically, there has been consensus that the ecological validity of developmental theories and models is important (e.g., [2,3,4,5,6]). However, the majority of the developmental research still relies on lab-based research, on the assumption that the lab phenomena resemble those encountered in the real world. Recent evidence indicates that often these assumptions can be wrong (e.g., young infants tend to have faces frequently in their view), and can have detrimental consequences for scientific progress [4,7]. While laboratory-based methods are of uttermost importance in testing with high precision the causal inferences and what could potentially happen in children’s lives if specific conditions or combinations of factors occur, they cannot show what actually does happen in everyday life [4]. For this purpose, the recommendation is to take a *naturalistic approach* and use methods that capture the rich diversity of a child’s spontaneous responses in their natural environment as well as the distribution of children’s experiences (e.g., [2,3,4]).

For the past decade, there has been a sustained effort towards increasing the use of naturalistic methods (e.g., [8,9,10,11,12]). These efforts confirmed on the one hand that the naturalistic approaches are much needed, but on the other hand they also revealed that, to a great extent, the necessary tools are massively lagging behind (e.g., [13]). Traditionally, naturalistic methods were predominantly focused on observations of behavior and environmental factors conducted by a researcher physically present in the infants and children’s environment, sometimes equipped with a video camera or an audio recorder. While useful in capturing some aspects of children’s behavior and the aspects of the environment to which they may be related, these approaches do not provide the degree of precision and sensitivity necessary for capturing the complexity of the mechanisms supporting the wide diversity and quickly changing behaviors, cognitive and emotional abilities. Furthermore, they lack the ability to densely capture with precision the dynamic changes in the auditory–visual input that are likely to contribute to infants’, toddlers’ and children’s cognitive and emotional development. Through the actual presence of the researcher, these approaches also tend to be fairly intrusive and change the environment. In this paper, we present the design, implementation, and validation of a platform (*EgoActive*) with integrated wireless wearable sensors and associated software aiming to overcome this major methodological limitation.

### 1.1. Importance of Wearable Sensors for Developmental Research in the Wild

Development is the result of many nested processes that take place and interact with each other over multiple time scales (e.g., [11,14,15,16]). In order to explain the complexity of developmental processes in the real world, technologies are required that can capture the emergence of a wide array of cognitive and socio-emotional functions, motor development, as well as the recurrent mutual interactions with the internal and external factors relevant for their emergence. Particularly relevant are technologies that do not rely on elaborate motor and language modalities of response, and can be easily deployed in the natural environment, with little interference to everyday life. To a large extent, the existent theories of development are predominantly relying on data from Western, Educated, Industrialized, Rich and Democratic (WEIRD) countries, and very little is known about the extent to which these models explain the socio-emotional and cognitive development of children worldwide (e.g., [17,18,19]). From this perspective, the technologies required for the naturalistic approach need to be scalable, and easy to deploy in a wide range of cultural and socio-economical environments. The EgoActive platform proposes to integrate measures of autonomic nervous system (ANS) function, in particular measures of cardiac activity, and body movement, with measures of the visual and auditory environment as it appears in infants’, children’s and caregivers’ egocentric perspectives. 

The motivation for measuring the dynamic patterns of the ANS rests on the fact that it is one of the outputs of the central nervous system [20,21] which underlies many behaviors, from emotional expressions, to vocal productions [21,22,23,24,25]. ANS activity measurement has been fruitful in understanding both typical and atypical development, with atypical ANS shown in autism manifestations of spectrum disorders [26,27], attention deficit and hyperactivity disorders (ADHD, [28]), conduct disorders [29], as well as the emergence of other neuropsychiatric conditions [30]. Therefore, towards our aim of developing technologies that can accurately capture the complex and multifaceted nature of the developmental process occurring in the natural environment, measures of ANS activity are an ideal candidate. 

Amongst the many ANS indices, of high importance are the demonstrated links between specific patterns of heart rate (HR) changes and cognitive functions, such as attention (e.g., [31,32,33,34]), as well as changes in arousal and emotion regulation abilities [35,36]. For example, during periods of sustained attention, the HR registers increased deceleration in tandem with overall quietness of the body movement [37,38,39,40,41,42]. Higher HR deceleration during sustained attention is associated with less distractibility [43,44] and enhanced neural processing of the attended information (e.g., [45,46]). Attention is a crucial cognitive function which registers rapid developments during the first year of life. It is essential for many adaptive processes throughout the lifespan, as well as a building block for the development of many complex cognitive abilities, such as the executive functions (e.g., [47,48,49,50,51,52,53,54,55]). The early development of attention as well as the cognitive functions it supports, set the infants to fare better in many aspects of life in subsequent years (e.g., [56]). 

The specific variations in the HR which occur as a function of the respiration cycle under heavy control from the parasympathetic nervous system (respiratory sinus arrhythmia (RSA), [57]) are also important for understanding development [58,59,60]. For example, accumulating evidence suggests that individual differences in children’s baseline RSA are associated with their emotion regulation abilities (e.g., [58,61,62,63,64,65,66,67,68]) and the quality of social interactions [69]. Importantly, measures of infant and children’s baseline RSA are sensitive to environmental factors, such as caregiver’s mental health, and caregiving behaviors (e.g., [61,70]), and are predictors of several developmental outcomes. Feldman et al. [71] have found that infants with high baseline RSA manifest attenuated stress response, have more organized sleep and better cognitive control at the age of 10-years [71]. On the other hand, low baseline RSA has been linked to the emergence of anxiety disorders, aggression (e.g., [58]), and oppositional defiant and callous-unemotional behaviors [72].

Alongside measures that can provide insights into different internal cognitive functions and affective states, it is also very important to measure the environmental factors and the diversity of experiences that contribute to their development [73,74,75,76,77]. Research within the last decade has shown that the visual and auditory events that occur in infants’ and toddlers’ egocentric perspectives are dramatically different from what adults experience [7,78,79]. Furthermore, what infants see from their own perspective changes dramatically throughout the first years of life, with various factors contributing to these differences. As developing organisms, infants tend to actively seek the information required for their further development [80], and thus their sensory and cognitive abilities at different points during development influences the type of environmental information they can attend [80,81]. Infants’ socio-emotional and cognitive development is also likely to change the characteristics of their environment. Extensive research shows that adults modulate their facial and vocal expressivity towards infants to match their sensorial and cognitive abilities, in order to facilitate the processing of relevant information [82,83]. Infants’ own motor development also influences the environmental information they can access [7,84]. As infants gain motor independence, from being able to maintain a stable head position to crawling and walking, visual and auditory objects, including people, will be perceived from more varied angles and distances [7,84,85]. Head-mounted cameras have been shown to be ideal for capturing the diversity of visual and auditory information that appears in infants’ and toddlers’ views in the natural environment [7,73,78,79], as well as how this changes at different time scales, from variations during short term activities, to changes throughout the day, weeks and months. For example, in their seminal study using head-mounted cameras in the ‘wild’, Jayraman and colleagues [78,79] have shown that while human faces are more frequent in young infants’ views, hands and other parts of the people’s bodies are more often contained in the environmental scenes available to older infants and toddlers.

### 1.2. Limitations of Existing Wearable Sensors for Developmental Research in the Wild

For the last decades, the advances in consumer-directed wearable biosensing devices have shown that the measurement of egocentric views, cardiac activity, and body movement in the wild is not only possible, but that the general public is open to adopt such technology for everyday monitoring of physical health and activity (e.g., [86,87,88]), but also in various professional settings (e.g., [89,90,91]). Although such technology has been predominantly developed for adults, its ubiquitousness suggests that similar technologies for infants and young children could be received with a fair degree of openness for researching development in the ‘wild’. However, creating wireless wearable solutions for developing populations, particularly for research in the ‘wild’ without the direct supervision of a specialist, presents important challenges. Flexibility, safety, unobtrusiveness and accuracy are some of the key challenges. 

Many of the previously developed wearable devices, both head-mounted cameras and body sensors, that have the necessary accuracy to be used for research purposes involve wires, are fairly bulky, and are difficult to operate by a non-specialist. For instance, in terms of recording the cardiac activity and body movement, options such as those created by Biosignalsplux (PLUX Biosignals, Lisbon, Portugal) and Biopac (BIOPAC Systems, Inc., Goleta, CA, USA) involve wet electrodes connected by wires to a data acquisition hub. The hub in itself can be fairly bulky and heavy, particularly for the younger infants which interferes with their body movement, and together with the wires present safety issues and are intrusive for everyday routines. Most of the head-mounted cameras present similar issues (e.g., [9,92,93]). For instance, the solution presented by Long et al. [9] involves the need of a bulky helmet and fairly large GoPro Hero 10 Bones camera (GoPro Inc., San Mateo, CA, USA). Many of these devices, particularly those for recording cardiac activity and body movement, are also usually difficult to operate by a non-specialist [13] which makes long term deployment in the natural environment impossible or extremely difficult. Other options, such as the sensing vest created by Maitha et al. [13], although it is largely wireless and has been described as easy to operate by non-specialists, is fairly heavy for young infants (i.e., it weighs ~400 g). The vest also contains to a large extent rubber, it covers most of the infant upper torso, and it needs to be fitted pretty snugly around the body for good signal quality [13]. This leads to overheating, which is a significant issue for its use in warmer climates or where air conditioning is not available. Therefore, it is not ideal for being deployed at scale in a wide range of socio-economical environments. A limitation of many of the head-mounted cameras used for research in the ‘wild’ that are on the lighter side is the fairly narrow field of view (e.g., Looxcie-69 × 41° [H × V]; Looxcie, Inc., Sunnyvale, CA, USA), which limits the accuracy for capturing what is likely to be visually fixated by the wearer. They also tend to have fairly poor video resolution (e.g., 720 × 480 pixels) which creates difficulties for automated methods for extracting the relevant data [9,92]. This is particularly important since dense sampling of naturalistic experiences leads to big datasets which cannot be analyzed without automated algorithms for meaningful data extraction (e.g., [94,95]). Importantly, none of these devices, usually advertised as spy or active cameras, have been specifically designed for infants and young children, and require custom mounts to be worn by developing populations (e.g., [9,92,93]). They also have a very limited battery life, usually under an hour, and hence do not allow uninterrupted dense data recording.

For recording cardiac activity and body movement, commercially available wireless options, such as the Gabi Smartcare armband (Gabi Smartcare, Belgium), have been specifically designed for young infants, and more easily meet the criteria for wearability and unobtrusiveness. However, many of these options rely on photoplethysmography, which is prone to noise caused by motion, environmental light, loose contact with the skin, and also poses issues for dark skin (e.g., [13,96]). In many cases, such as the Gabi Smartcare armband (Gabi Smartcare, Belgium), the devices are not validated on infants and young children during active states [97]. Furthermore, many of these commercially available options tend to have the raw signal and the data storage under a paywall. This limits their affordability, makes it difficult to correct and verify the data, and also difficult to test new developments in the raw signal processing [13]. 

Crucially, to our knowledge, there are no technological solutions that allow the synchronized recording of the egocentric view and autonomic activity across multiple individuals in the ‘wild’. The integration and joint analysis of these streams of data are essential for understanding the complex and recurrent mutual interactions between the development of infants’ cognitive and socio-emotional functions on the one hand, and the environment experienced by them on the other (e.g., [34,98,99]). 

### 1.3. The EgoActive Platform

In the following sections, we present the design, implementation and validation of the EgoActive platform, which addresses many of the limitations of wearable technologies for developmental research.

The process of designing the platform was guided by a series of high-level requirements prescribed by the aims of the ecologically valid developmental research approaches, as indicated above. More specifically, we focused on the following: to be suitable for infants, young children, and individuals throughout the lifespan; to present the robustness and precision required for scientific research; to be able to temporally synchronize streams of data generated by multiple devices and individuals; to be scalable to a wide range of socio-economical and geographical environments, and affordable; to be easy to use by individuals with a wide range of expertise, from tech-savvy to those with very little experience of using technical devices; to have an open design and access to raw signal and data in order to maximize its use and further development by the scientific community. 

In order to satisfy these high-level requirements, we identified a number of more specific physical and operational requirements that guided the implementation of the design.

Physically, we require that the wearable devices are comfortable and of sufficiently low weight that an infant is largely unaware they are wearing them and their behavior is unaffected. The devices must be safe to ensure that no participants are harmed by taking part in data collection activities and to meet regulatory requirements. They should be unobtrusive, i.e., small and as out of sight as possible when worn, such that participants behave naturally and do not react to the presence of the devices on other participants. They should be easy to manufacture, using off-the-shelf parts and widely available materials as far as possible, in order to keep costs low and simplify the process of other researchers replicating their construction.

There are also requirements relating to the operation of the devices and system as a whole. Since the data capture process requires significant time investment from the participants and the data itself has high scientific value, the robustness of the data storage is paramount. We therefore require robustness and redundancy in data storage in order to minimize data loss. This is reflected in the inclusion of a data backup capability within our system and to minimize data loss when wearable devices are turned off or lose power during recording. In order to minimize how often the participants must recharge or switch devices, we require wearable devices to have extended continuous recording capability both in terms of battery life but also the capacity of data storage media within the devices. The resolution, temporal for body sensor and spatio-temporal for cameras, must be sufficient to capture the events of interest. For the body sensor, this relates to the rate of change in heart rate during attention onset and offset. For the camera, the spatial resolution must enable extraction of visual information comparable to that extracted by the participant’s visual system. There is a related requirement that the field of view of the camera should be adequate to capture all, or the majority, of the scene contents to which the participant’s visual system is attending. We also require that the time series data collected from all devices is temporally aligned. This necessitates some form of temporal synchronization procedure between devices. Our goal is to retain the simplicity of the devices by minimizing addition of extra sensors for synchronization (utilizing existing sensing capability where possible) and for the synchronization process itself to be simple to execute without errors. Finally, we anticipate the devices collecting significant volumes of data. If a single participant collects tens of hours of video data, this quickly becomes challenging to store, manage and process. Our ultimate operational requirement is that the processing required to transform the raw data into usable time series is automatic and computationally efficient, therefore successfully scaling to big data.

In Section 2, we present the design and implementation of the hardware and software parts of the EgoActive platform. The hardware comprises three parts: (1) charging, synchronization, and data back-up station; (2) head-mounted cameras (HMCs); and (3) ECG and acceleration body sensors. In Section 3, we present the software to support different functions of the hardware, and to implement signal preprocessing steps required prior to the extraction of more meaningful features. Specifically, the software we developed comprises of: (1) Android application for device synchronization and temporary data back-up; (2) open-source software for extracting and processing the synchronization codes from the audio-video and ECG time series, as well as for temporally aligning them; (3) open-source software for extracting HR from the raw ECG signal; and (4) open-source software that automatically detect which portions of the video and ECG data are of sufficient quality to be usable.

## 2. Platform Hardware Design, Fabrication and Validation

### 2.1. Overview

The sensing part of the EgoActive platform was developed in order to allow recording for extended periods of time of the egocentric perspective of wearers (infants, children, and their caregivers), temporally aligned with measures of cardiac activity (i.e., ECG) and body movement in the natural environment.

**Figure 1:**
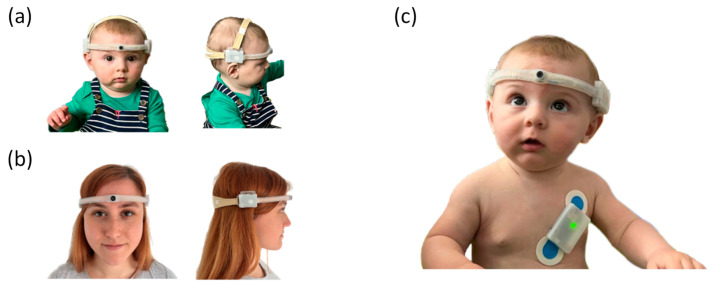
The EgoActive head-mounted camera (HMC) worn by a 6-month-old infant (**a**) and an adult (**b**). The HMC dimensions are tailored to fit the head circumference of young infants (6- to 8-months-old), older infants and toddlers (9-months-old and older), and adults. The HMC for younger infants integrates a narrow-FOV lens, while the HMC for older individuals integrates a wide-FOV lens. In all cases, the HMC is very light (52 g for the narrow-FOV version, and 58 g for the wide FOV). (**c**) HMC and the EgoActive body sensor worn together by a 6-month-old infant.

Towards these aims, the sensing part of the EgoActive platform includes wireless head-mounted cameras (HMC), with different options for the lens field of view (FOV), that can be worn simultaneously by infants, children and adults (Figure 1).

**Figure 2:**
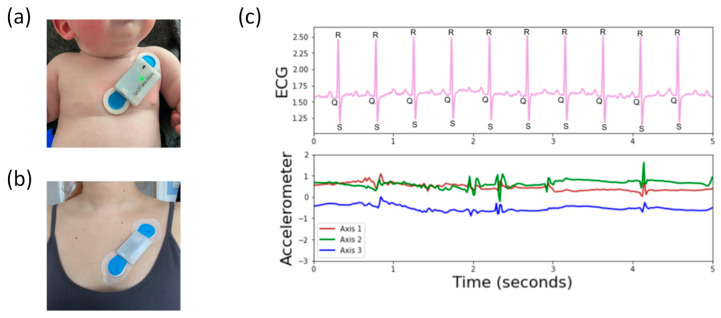
EgoActive body sensor positioning on a 6-month-old infant (**a**) and an adult (**b**). The EgoActive body sensor has a small footprint and it is light. (**c**) A sample EgoActive body sensor recording of an infant. The ECG and three-axis accelerometer signals are recorded simultaneously (at 250 Hz for the ECG, at 65 Hz for the accelerometer). The QRS complex in the ECG can be seen clearly repeated in this signal. While the precise morphology of the QRS complex can vary (e.g., depth of the Q and S troughs), the R-peak is a consistent feature necessary for later analysis.

For recording cardiac activity and body movement, the EgoActive platform includes integrated wearable body sensors that record ECG via wet electrodes and body movement via a triaxial accelerometer (Figure 2). The integrated sensor also includes a photo sensor used for temporal synchronization with other devices in the platform (e.g., HMC).

The dimensions of the devices are tailored to fit the head and body size of infants of different ages, as well as older children and adults. When used concomitantly, the devices have continuous recording autonomy of up to 2 h 10 min, which facilitates habituation to the device and spontaneous behavior. With the inclusion of multiple devices for each individual, the platform also gives the possibility to record continuously for longer than 2 h 10 min by swapping devices when the battery runs out. 

The devices can be used either independently, or related to each other. For the more complex scenarios where the research questions require the analysis of the data recorded by the head-mounted camera to be temporally aligned with that recorded by the body sensor (from one or multiple individuals), the precise temporal cross-device synchronization is a major challenge. Cross-device synchronization entails sensing a signal from a shared source on all devices. Our devices sense different modalities of data: the camera captures video and audio while the body sensors capture ECG and acceleration. This presented two options. One was to augment one of the devices with an additional sensor such that a single synchronization source could be used by all devices, the other was to use a different synchronization source for the two types of device (e.g., vibration for the body sensor and sound for the camera). We chose the first option, more specifically to use a coded light signal. This can be recorded directly by the cameras while we augment the body sensors with a light sensor. This adds very little to the cost or complexity of the body sensor and we store only a binarized light signal adding very little to data storage requirements. This solution simplifies the generation of the synchronization signal and reduces a possible source of error by avoiding the need to ensure the two synchronization signal sources are themselves synchronized. 

**Figure 3:**
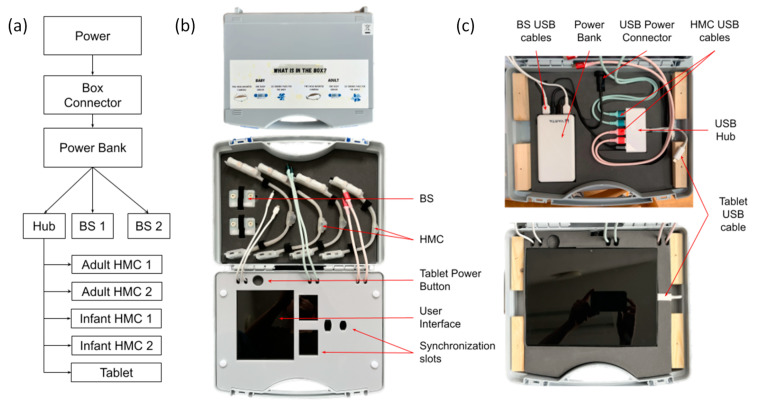
The EgoActive platform: (**a**) Schematic diagram of the connections between components. (**b**) Display of platform with components including the body sensors (BSs), head-mounted cameras (HMCs), and the electronic devices and accessories involved in synchronization, data back-up, and charging. (**c**) Arrangement of component connection.

The functioning of the sensing hardware components is supported by a base unit that implements their synchronization, charging, and temporary data backup. Figure 3 illustrates the base unit with its components and the connectivity diagram. All components are compactly contained in a suitcase style box, in a layout that is intuitively accessible to a wide range of users. The key elements of the base unit are represented by the Android Samsung A8 tablet (Samsung Electronics Co., Ltd., Suwon-si, Republic of Korea) and the powerbank. The tablet’s main functions are to support the temporal synchronization of the head-mounted cameras and body sensors, as well as the temporary data back-up for the duration of deployment in the natural environment via a custom Android application (see Section 3).

### 2.2. Head-Mounted Camera

The design goals for the head-mounted camera were to produce a device able to record visual scenes from the wearers’ egocentric perspective, as well as the associated sound for an extended continuous recording. In order to capture most visual information that the wearer is likely to fixate, the captured visual scenes were required to approximate as much as possible the human FOV. In order to meet our criteria for comfort, safety, and unobtrusiveness, the physical device was required to be small, lightweight, and to adapt to the differences in head circumference across different age groups. Of particular importance was to achieve a small footprint relative to the small head of infants given that all previous head-mounted cameras used for research with infants are large [9]. We also aimed to achieve a minimum 2 h of battery life that can support continuous recording without the need to operate the device. Alongside comfort and small footprint, this would ensure that once the camera is placed on the head and recording, the wearer will habituate to it and his/her behavior will be less likely influenced by it. 

**Figure 4:**
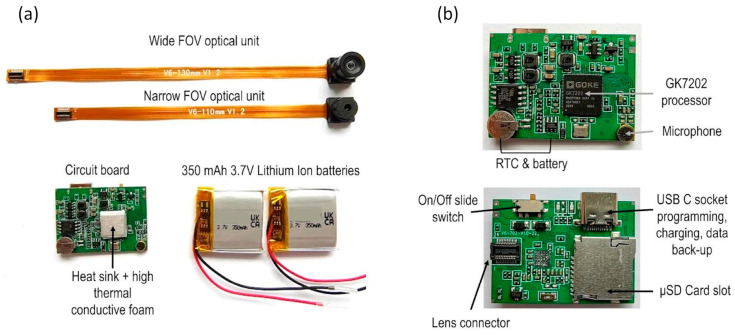
(**a**) The HMC circuit board with the optical units (narrow and wide FOV), and the 350 mAh 3.7 V lithium ion batteries; (**b**) the circuit board and its key elements. The circuit board measures 35.00 mm in length, 25.00 mm in height and 0.50 mm deep.

These requirements posed many challenges, which largely stem from the need of achieving a small footprint for the entire device and comfort to wearer, and the fact that the majority of the operational requirements involve large components. Many of the existing head-mounted camera designs involve containing the optical units/lenses, circuit board and battery in a single case. This tends to lead to a fairly bulky device, which often is difficult to align with the wearer’s line of sight. These bulky devices (e.g., GoPro Hero Bones (GoPro Inc., San Mateo, CA, USA), Looxcie (Looxcie, Inc., Sunnyvale, CA, USA), and Veho Muvi Pro (Veho, Southampton, UK)) need a special head mount and tend to be worn fairly high on the forehead and head. Through their sheer size, they can represent a safety risk for younger children, and are a major intrusion to the appearance of both children and adult wearers. We therefore distributed the components (circuit board, lens, battery) alongside a head-band like mount, which can wrap around the wearer’s forehead. In this design, the circuit board occupies the flat surface above the right ear. We further used two smaller batteries located above the left ear, rather than a single large battery, which allows the headset to flexibly fit around the head but allows for extended continuous recording. This distributed design not only led to a smaller footprint of the parts that are directly in sight, but also a better alignment of the lens with the center of the wearer’s FOV (Figure 4).

#### 2.2.1. Hardware Design

**Circuit board.** The circuit board was specifically customized to meet the functional and operational requirement by an external company. It was developed around a JX-F23 2.0 MP image sensor with MIPI CSI2 and dual-data lane serial interfaces (Silicon Optronics, Inc., Shanghai, China). The JX-F23 consists of 1932 × 1088 active pixel sensor array, where each pixel is 2.8 × 2.8 μm, with on-chip 10 bit ADC, programmable gain control, and correlated double sampling in order to reduce fixed pattern noise. The sensor has an electronic rolling shutter, sensitivity of 3300 mV/lux-s, and RGB Bayer pattern for the color filter array. The image sensor is coupled with a Goke GK7202 processor (Goke Microelectronics Co., Ltd, Changsha, China). GK7202 has low power consumption, supports multistream encoding capabilities, and efficient video compression ratio. Prior to video encoding, frames at full HD (1920 × 1080 pixels) resolution are center-cropped from the pixel data recorded by the sensor. The video encoder uses a variable frame rate which averages 30 frames per second. 

The circuit board also integrates a real-time clock (RTC) which enables the alignment of the entire platform to the actual time via a temporal synchronization signal described in Section 3.1.1. The RTC can be set by a text file in the SD card. In order to maintain the continuous function of the RTC, the circuit board was designed to include a 3 mAh rechargeable lithium-ion coin battery exclusively dedicated to power the RTC. Whenever the camera is charged, the RTC battery will be recharged as well. For audio recording, the circuit board also integrates a microphone. Audio is recorded in stereo at 32 KHz and 32 bits per sample and encoded as MPEG AAC audio.

**Optical unit(s).** Two optical units/lenses were considered for the head-mounted camera, one which has a wide field of view (FOV, diagonal 106.6°, horizontal 89.3°, vertical 58.1°) and one with a narrower FOV (diagonal 73.4°, horizontal 62.8°, vertical 38.0°). FOV was measured via a geometric calibration performed using the Matlab (The MathWorks, Inc., Portola Valley, CA, USA) Camera Calibrator tool (see Appendix B for additional details). While the wide FOV captures a larger part of the natural infant and adult FOV, it is physically fairly large, which would lead to a forehead section that is bulky relative to the small head dimensions of infants, particularly the younger ones (5–6 months-old). The wider FOV also exhibits more significant fisheye distortion (larger radial distortion parameters in intrinsic calibration parameters). If required for subsequent processing, the calibrated distortion parameters can be used to undistort the images prior to processing. A larger headset would be more obtrusive, and also potentially pose safety risks. In order to overcome these limitations, we chose to evaluate a smaller FOV optical unit as well. The validation studies we conducted (see Section 2.5) indicate that the narrower FOV lens reliably captures the majority of the fixations made by the 6-month-old infants, while the wider FOV is required for a similar performance for older infants, children and adults. In light of these findings, it was decided to design versions of the head-mounted camera that include the narrow FOV for the younger infants (6–7 months), and the wider FOV for older infants (>7 months old), children and adults. The lenses are connected to the circuit board via a surface mount, flexible printed circuit (FPC) connector.

**Figure 5:**
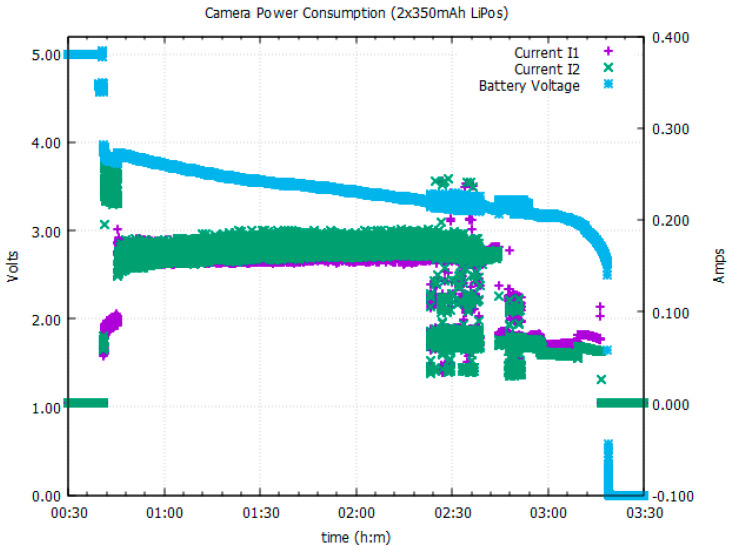
The HMC power consumption showing a steady current consumption of 160 mA per battery for nearly 2 h.

For both types of lenses, the HMC device has an average current consumption of 320 mA at 3.7 V supply potential. In order to enable a minimum of 2 h continuous recording as per our requirements while maintaining a flexible and small footprint, two fairly small 350 mAh lithium ion batteries (24.0 × 25.0 × 6 mm, 3.7 V 350 mAh) were connected in parallel. The voltage versus current curves (Figure 5) show how the 2 batteries share the load evenly during both the recording and charging cycles, supplying approximately 160 mA each during the camera recording period and for over 1 h 40 min with the measurement equipment connected. However, when disconnected from the test equipment, a fully charged 700 mAh battery pack was observed to keep the HMC actively recording for approximately 2 h 10 min. 

**Video format.** Video data are written to a 128 GB micro SD card in 5 min consecutive blocks. After recording, these blocks can be stitched into a continuous video of the full session length. In the event that the power is removed by switching off the device then any data held in the device’s internal memory will be lost. Therefore, in the worst case, up to 5 min of recording could be lost. In the context of extended real-world recordings, this was considered to be a reasonable tradeoff. In practice, however, temporary files are written while the 5 min block is being recorded. If power is lost and recording later resumed, this partial block continues to be written to until 5 min of video has been recorded. We can automatically identify these split blocks by finding consecutive frames with large gaps between their presentation timestamps. We can split the video file at this point and restore the partial block to the end of the previous session. The videos are stored in MP4 format, compressed using the H.264 codec with a 1920 × 1080 pixel resolution, and MPEG-AAC codec for audio with stereo channels, sample rate of 32 kHz and 32 bits per sample.

**Temperature management.** For a wearable device, the temperature is relevant for comfort and safety. We aimed at maintaining the temperature of the functioning device below 43 °C in line with the more conservative safety standards for audio/video information and communication technology equipment (BS EN IEC 62368-1:2020+A11:2020). Towards this aim, we designed a temperature management system. First, the microprocessor was located on the external surface of the circuit board (facing away from the body). Second, to facilitate the dissipation of the temperature away from the body, an aluminum heat sink was attached with a layer of high thermal conductivity foam (5 W/m-K) on the surface of the microprocessor, while the internal side of the circuit board (facing the body) was covered with a layer of thermally insulating foam (0.8 W/m-K). In addition, the liquid silicone wrap of the casing (see Section 2.2.2) was intended to add an extra layer (1 mm thick) of thermal insulation at the point of contact with the body. 

**Figure 6:**
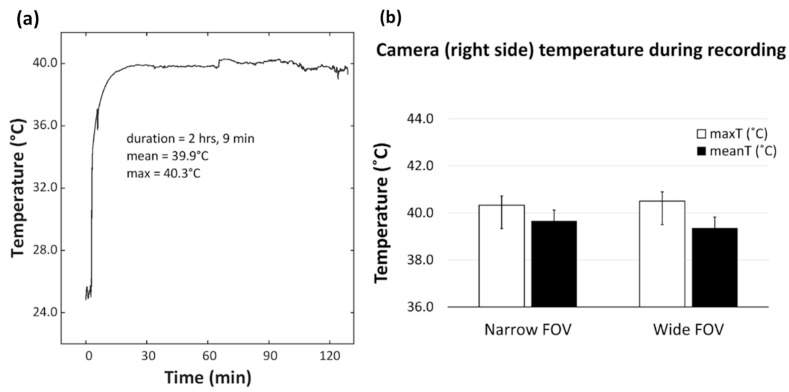
The HMC temperature overtime: (**a**) An example temperature recording (2 h, 9 min) for a narrow-FOV camera. The maximum and mean temperature was calculated from 10 min from recording onset. (**b**) The average maximum and mean temperature for narrow-FOV (*N* = 5) and wide-FOV (*N* = 5) cameras. Error bars represent the standard error of the means.

To test the efficiency of this system, the temperature sensor from Biosignalsplux (PLUX Biosignals, Portugal) was used to measure temperature of the HMC headset section containing the circuit board at the point of contact with the skin. We chose to measure the temperature at this location given that it is the only part likely to record changes in temperature during functioning. The temperature sensor was a NTC thermistor element (2.04 mm diameter) which has an operational range between 0 °C and 50 °C. We measured the temperature of both the HMC headsets incorporating the wide (*N* = 5) and narrow (*N* = 5) FOV lenses. The temperature was recorded continuously for a minimum of 1 h 50 min, while the camera was worn on the head by an adult as it would typically be for research purposes, at an average room temperature of 25.29 °C (*SD* = 0.60 °C). The average recording length (time on) was 118.0 min (*SD* = 17.2 min). Both the HMC with the wide- and narrow-FOV lenses recorded an average temperature below 40 °C (Figure 6), hence within approximately 3 °C of the usual body temperature.

#### 2.2.2. Headset Case Design

The headset case design was one of the most challenging aspects of the platform to achieve. The housing of the circuit board, optical unit, batteries, and wiring was required to be both rigid and flexible. Rigidity was needed to protect the batteries, circuit board and optical unit, whilst flexibility was a must for allowing the entire setup to naturally follow the contour of the head and to be comfortable when worn. Furthermore, the materials had to be adequate for extended contact with the skin. Particularly for the young infants, comfort and the possibility to wear the HMC in contact with the skin were of uttermost importance. Discomfort during psychophysiology recordings is well known to lead to a high attrition rate in infancy research [100,101,102,103]. 

**Figure 7:**
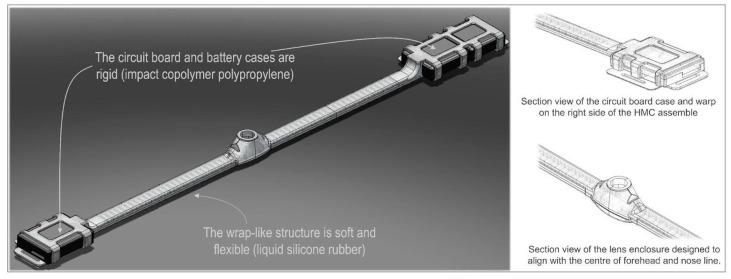
The EgoActive HMC casing relies on a 2-part design that combines rigid and flexible materials in order to satisfy the mechanical properties imposed by the requirements. The wrap-like structure is made of soft and flexible liquid silicone rubber, while the batteries and circuit board cases are made of rigid impact copolymer polypropylene. The entire camera, electronics and casing, weighs 52 g for the narrow-FOV lens version and 58 g for the wide-FOV lens version. The lens enclosure measures 8 mm in height for the wide-FOV lens, and 4 mm in height for the narrow-FOV lens. The depth of the circuit board and battery casings with the silicone wrap is 12 mm.

In order to satisfy the mechanical properties imposed by our requirements, we decided to adopt a 2-part design strategy that combines rigid and flexible materials. Within this new innovative design (Figure 7), the battery and circuit board are housed in rigid casings made from a thermoplastic material, which is further enclosed by a web-like casing made from an elastomer. The wrap-like casing acts as a carrier to combine all the electrical components, housing the cabling, battery, circuit board, and optical unit. The design feasibility and material compatibility were verified using 3D printing. For the rigid cases, 3D printing via Multi-Jet Fusion was used because it produces functional, end-use production parts that would enable us to have true verification in terms of mechanical properties and design feasibility. Multi-Jet Fusion uses an inkjet array to selectively apply fusing and detailing agents across a bed of nylon powder, which are then fused by heating elements into a solid layer. For testing the prototypes of the wrap-like casing, Polyjet 3D printing with digital photopolymer was used. The Polyjet 3D printing builds multimaterial prototypes with flexible features which works well in simulating components made to have elastomeric features. It uses a jetting process where voxels of liquid photopolymer are sprayed from multiple jets onto a build platform and it is cured in layers that form elastomeric parts. In our particular case, the material of choice was 3DP silicone because it has similar characteristics to the true liquid silicone rubber (LSR), such as reproducibility after deformation or stress and elasticity. 

The final design encloses all unnecessary access points, except the USB charging socket, SD card, the on/off switch, and the lens. The design also includes a recess for the SD card to prevent it from accidentally being ejected while still maintaining the ability to remove the card for reading its data; a recess for the slide switch to prevent any small parts breaking away and becoming exposed outside the case.

For the final manufacturing of the HMC headset case, we used plastic injection molding. For the circuit board and battery cases, an impact copolymer polypropylene was chosen. This material exhibits high melt flow rate which allows it to achieve a very thin casing, whilst also having very high impact resistance and very good thermal stability. As a specific material type and brand, we chose INEOS PP 500-GA20 (Ineos, UK). According to the manufacturer’s data sheet, this material is recommended for toy manufacturing, food containers, and safe to be in contact with human skin. It is also widely available. For the web casing, we used LSR at ShoreA 60. During prototyping, we established that Shore 60 balances well between the requirement of having a soft and comfortable headset, and the ability to maintain the shape required for accurate positioning on the head. Furthermore, LSR is also known to have electrical and heat insulating properties, which are relevant in terms of safety and comfort. As a specific material type and brand, we chose Elastosil 3003 (Wacker Chemie AG, Germany) (Shore 60 A/B) because, according to the manufacturer’s datasheet, it has a good biocompatibility profile, it is recommended to be used for products that are in extended contact with infant and adult skin, and for products that are in contact with food. The PM-T2 finish gives a pleasant soft touch feel.

The headset is held in position on the infant head by two straps. For both the infant and the adult, the back strap adjusts the length of the entire headset to the individual’s head circumference. For the infant, the top strap is primarily meant to prevent the headset sliding down while in use. The straps are manufactured from a soft elastic neoprene material. Due to the use of LSR Shore 60, which is flexible but maintains shape, neither the infant nor the adult headset require a tight fit around the head in order to maintain the desirable location of the lens (i.e., above the eyebrows and approximately aligned with the nose).

### 2.3. Body Sensor

**Figure 8:**
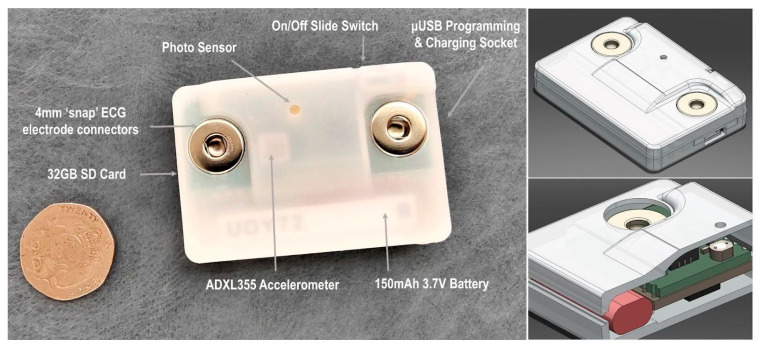
EgoActive body sensor measures 54.00 mm in length, 36.80 mm in height and 11.90 mm deep, while weighing 21.0 g.

The design goals for the body sensor were to produce a small and lightweight device able to record reliable heart rate and body movement data over a period of 3 to 4 h (Figure 8). The recorded data would need to be time coded in such a way that it could be accurately synchronized during post-processing to a video recorded using a HMC. It should also be low cost, comfortable, safe, meet relevant regulations and be easy to reproduce on a small-scale production run. 

#### 2.3.1. Hardware Design

The body sensor is built around an Adafruit Feather M0 Adalogger^TM^ (Adafruit Industries LLC, New York, NY, USA) which features a ATSAMD21G18 ARM Cortex M0+ (Microchip Technology Inc., Chandler, AZ, USA) low power embedded microcontroller, running at its default system speed of 48 MHz. The Adalogger (Adafruit Industries LLC, New York, NY, USA) provided a ready made solution for basic SD card interfacing and USB battery charging circuits. The Feather’s small form factor provided the perfect size, weight and processing power as well as having a library of support functions, available within the firmware development toolset, for logging data to an SD card. 

**Figure 9:**
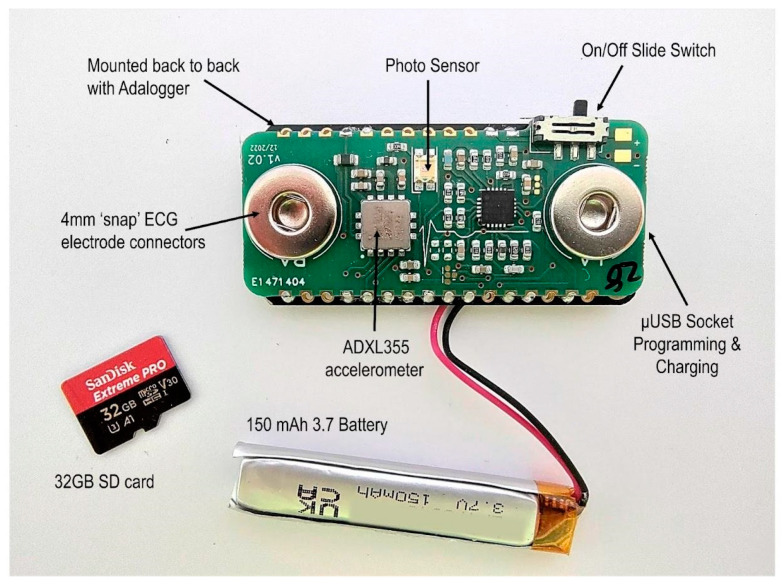
EgoActive body sensor circuit board mounted on the back of an Adafruit Adalogger^TM^.

To complement the Adalogger^TM^ (Adafruit Industries LLC, New York, NY, USA), a bespoke featherwing-compatible circuit board was designed (Figure 9) which contained the interface from the body measurement sensors (ECG, movement) and a photo sensor back to the Adafruit Feather M0 Adalogger^TM^ microcontroller (Adafruit Industries LLC, New York, NY, USA).

**ECG Interface.** For the ECG interface, an Analog Devices AD8232 (Analog Devices, Inc., Wilmington, MA, USA) single-lead heart rate monitor preamplifier was selected because of its suitability in conditioning noisy biopotential signals. It can be configured for either two or three electrode placements, with the third electrode being used as a right leg drive amplifier to reduce the common mode rejection of the system. We chose to use the two-electrode configuration for the simplicity offered to the end user when positioning the device on the body. The circuit around the AD8232 allows for a simple two-pole high-pass filter to help block the DC component from the input signal while also allowing the lowest cutoff frequency. The AD8232 also includes a ‘leads off’ detection circuit whose output is read and logged along with the recorded ECG signal data and although it is not currently used it is available as a secondary check for ECG validation during post-processing. The ECG signal is sampled by the microcontroller’s analogue to digital conversion peripheral (ADC) at a rate of 250 Hz. The 250 Hz sampling rate for the ECG is recommended as the minimum to accurately capture the R wave [104] and also for more advanced heart rate variability analysis (including RSA) [105].

**Accelerometer.** An Analog Devices ADXL355 (Analog Devices, Inc., Wilmington, MA, USA) three axis accelerometer was selected to act as the body movement detection sensor. Although not the cheapest option, its low power, low offset drift, ultralow noise, 20 bit binary output conversion and simple digital serial peripheral interface made it an ideal choice for this application. The accelerometer’s three-axis output data are sampled and recorded by the microcontroller at a rate of 65 Hz. These calibrated three-axis signals are combined into a single resultant signal during processing. 

**Photo sensor for temporal synchronization.** The body sensor does not include a RTC implementation and therefore cannot record a unique time stamp either contained within the file structure or recorded within the data itself. In order to address the requirements for temporal synchronization, the body sensor contains a Kingbright KPS-3227SP1C (Kingbright, Taiwan) ambient light photo sensor whose output is digitally sampled at a rate of 250 Hz. This ambient light photo sensor allows recording a 10 bit coded light sequence provided by the Samsung Android tablet (Samsung Electronics Co., Ltd., Suwon-si, Republic of Korea) via the bespoke software application. During post-processing, this light signal is used to realign the time of acceleration and ECG signal to the RTC of the head-mounted camera. The photo sensor (luminosity), acceleration, and ECG signals are all recorded concurrently.

#### 2.3.2. Firmware Design

One of the design goals was that the final device supports 3 to 4 h of continuous recording. Power consumption and therefore battery life were an important parameter to measure during the design cycle and firmware development, which had to be embedded with the requirement of maintaining a small footprint and hence finding a small battery.

As with the HMC, the body sensor data are stored locally on a micro SD card and during the first iterations, the firmware was structured in a way which prioritized data preservation by storing each and every new data sample to one of two human readable text files on the SD card, one for heart rate measurements and the other for the accelerometer body movement measurements. However, running the firmware in this way resulted in the device consuming an average of 320 mA due to a nearly continuous SD card activity. This would have required a sizable 1.3 Ah (ampere-hour) battery to meet the 4 h usage time of the design specification which was not suitable in terms of the small footprint requirement. The ECG and ACC sampling rate was also limited due to the amount of time the processor needed to wait for the SD card’s internal processes to finish, and thus restricting the speed of the main processing loop.

The average current consumption was reduced dramatically to 40 mA by restructuring the firmware, writing data in 512 byte blocks and thus reducing the number of individual data transfers to the SD card. Data read from the sensor interfaces are held within the microcontroller’s internal memory until there are enough data to efficiently write a 512 byte block to an open file on the SD card. This stores both the ECG to a significance of three decimal places (*SD* = 3.297), and the accelerometer data to a significance of three decimal places (range: +2 g to −2 g). After the 5 min block of data has been transferred to the currently open file and the data transfer is committed, the file is closed and a new file is opened ready to accept further data.

**Figure 10:**
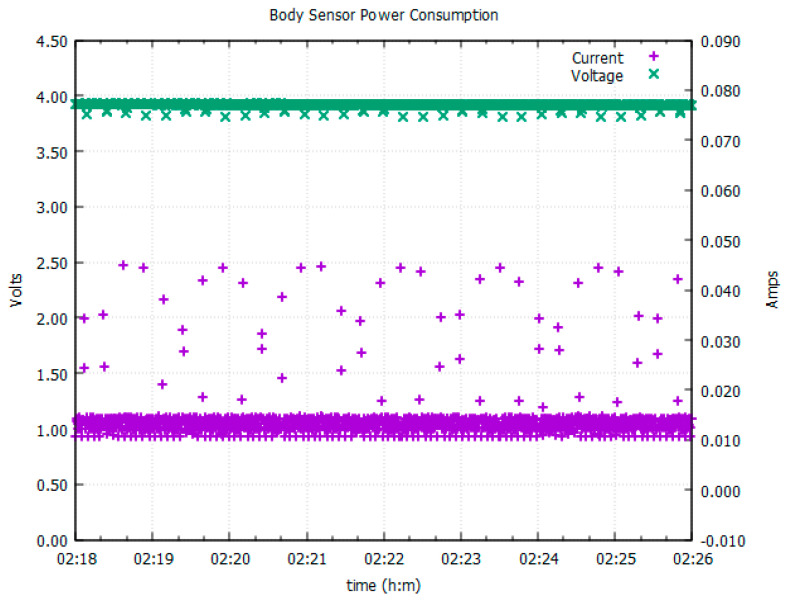
EgoActive body sensor mid-session power consumption, showing the battery voltage at 3.9 V with typical current consumption 12 mA (40.8 mA spikes).

Data transfer rates were improved further by storing the local data in a machine readable, raw format and off loading the process of converting the data into a human readable format to the post-processing scripts which run on more powerful desktop PCs (i.e., converting the .dat files to .txt files). Reducing the amount of time the device was waiting for an SD card data transfer and also removing the process of converting the raw sensor data to a human readable format meant that we could use this free time to increase the sampling rate of the ECG signal to 250 Hz. These efficiencies in data handling resulted in the device consuming only 12 mA average current (Figure 10), resulting in the selection of a fairly small lithium ion battery (46.0 × 9.0 × 4.5 mm, 3.7 V 150 mAh), meeting the requirement of a small footprint. The fully charged 150 mAh battery was observed to keep the body sensor actively recording for approximately 10 h.

With the efficiency gain from storing the raw sensor data, rather than human readable text strings, a 32 GB SD card can hold up to 4028 h worth of data in total. The data are stored in individual files holding 5 min worth of data at a time. In the event that the power is removed by switching off the device, then any data held in the devices internal memory will be lost; this would be a maximum of 5 min worth. In the context of the extended real-world recordings, this was considered to be a reasonable tradeoff.

#### 2.3.3. Case Design

The body sensor case was designed to meet the requirements of maintaining a small footprint for the device, comfort during use, protection of the electronics, and to provide user safety. Similar to the HMC, a prototype was designed, and the design feasibility and material compatibility verification was performed using 3D printing via Multi-Jet Fusion. The final small footprint design (Figure 7) allows minimal exposure of the device internal circuit components to the user. It encloses all unnecessary access points, except the USB charging socket, SD card and the on/off switch. The design also includes a recess for the SD card to prevent it from accidentally being ejected while still maintaining the ability to remove the card for reading its data; a recess for the slide switch to prevent any small parts breaking away and becoming exposed outside the case, and sufficient radius of external edges to achieve comfort.

In terms of the manufacturing of the final design, after factoring the casing design requirements in terms of material choice, quantity, and relatively low complexity; plastic injection molding was selected. For the material, an impact copolymer polypropylene was chosen (INEOS PP 500-GA20; Ineos, UK). This material exhibits high melt flow rate which allows it to achieve a very thin casing, whilst also having very high impact resistance and very good thermal stability. According to the manufacturer’s data sheet, this material is recommended for toy manufacturing, food containers, and hence safe to be in contact with human skin.

The mounting of the sensor on the human body is via the two ECG electrodes attached to the chest. We developed and tested our sensor design using the Ambu Blue sensors (Ambu A/S, Denmark) given their offset fitting that has been shown to reduce the signal noise during the data acquisition. The offset fitting also allows the sensor to be attached and reattached without the need to apply pressure on the body, which is particularly relevant for preventing infant distress. Furthermore, as per manufacturer’s instructions, these electrodes can be worn for extended periods of time. All these aspects are important in terms of achieving high quality data during recordings in the natural environment.

### 2.4. Base Unit 

The base unit provides a station with which to charge, synchronize, backup and transport the HMCs and body sensors. It comprises predominantly off-the-shelf hardware components, and a small proportion of custom made elements (e.g., the foam layer that maintains the electronics in place, the screen that covers the tablet). All components and materials are widely available, giving the possibility to anyone interested to build their own. We now describe the physical design and construction along with the specific hardware components used in the base unit.

The base unit is housed within a WAG TEKNO 2007 (W.AG Funktion + Design GmbH, Germany) polypropylene carry case (external dimensions: 340 mm × 275 mm × 83 mm). For further information regarding how different components are secured in the box, please see Section 3.1 and Appendix D.

We chose to use the Samsung A8 tablet for synchronization and backup purposes. The specific hardware model is important due to its ‘on the go’ functionality, which is required for the transfer of files between multiple external drives (the cameras) and internal storage via its USB-C port as well as simultaneously charging over the same port. It also supports large capacity SD cards (512 GB) which we use as the backup location.

Although the head-mounted cameras, body sensors, and tablet can be charged via regular USB cables from any power source, we designed the base unit to provide a backup power supply and also to act as a power distribution hub. The base unit includes a Varta 57977 15,000 mAh power bank (Varta AG, Germany) charged via the micro USB input, which is connected to a flush mount micro USB port in the back of the carry case, allowing power input from a USB AC adapter. Importantly, the power bank supports pass-through charging, meaning that it can be simultaneously charged and used, allowing the base unit to be left connected to mains power over the period of use. The power bank provides two USB-A outputs which are used to charge the body sensors. The power bank USB-C power delivery (PD) output is connected to the PD input of an RS PRO 4 Port USB 3.0 USB C Hub (RS Group plc, UK). This serves two purposes: first, it connects the Samsung A8 tablet (Samsung Electronics Co., Ltd., Suwon-si, Republic of Korea) to four USB-A cables to which the HMCs can be connected for data backup; second, it supplies power to the tablet and HMCs for charging. The power bank allows some autonomy from the main electricity source, which facilitates deployment in environments where electricity is scarce or inconsistent, and gives a certain degree of portability to the entire platform (e.g., can be used outdoors during a day out).

### 2.5. Validation and User Experience of the EgoActive Hardware

The criteria for the design of the EgoActive hardware were based on factors such as suitability for a wide age range (i.e., from infants to adults) and ease of use by families with varied technical skills. To ensure that these criteria did not impact the quality of the information recorded by the camera lens and ECG sensors, we conducted a series of validation studies that compared our sensors to other commercially available systems. Furthermore, to assess user experience, we recruited 7 families with 6-month-old infants from the UK to use the EgoActive Platform in their homes, while carrying out their daily routines and activities. The caregivers were asked to record approximately 4 h/day, both the infant and the caregiver simultaneously wearing the devices, for a period of one week. We used subjective reports from the caregiver to assess whether infants and caregivers had a positive experience wearing and operating the HMC and body sensor, and whether wearing both devices interfered with daily routines and activities.

The following sections present the results of the validation studies for the HMC and sensor, and users’ experiences of the EgoActive platform.

#### 2.5.1. Validation Studies of EgoActive Head-Mounted Camera

##### Suitability of the FOV

**Figure 11:**
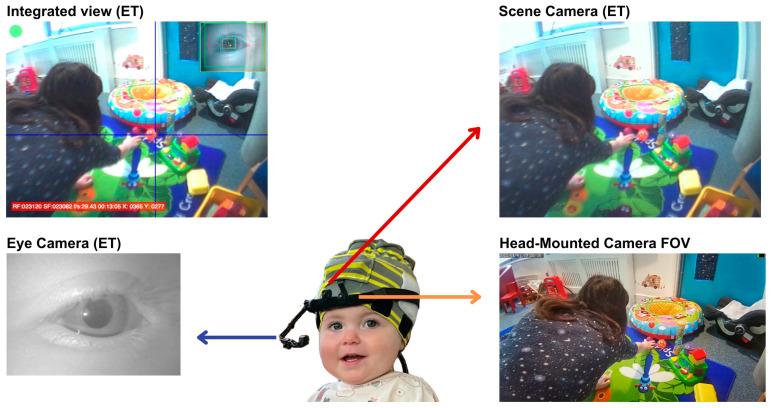
Positioning of equipment and visual example from the validation of the head-mounted camera (HMC) wide field of view (FOV) alongside the eye-tracking (ET) system.

We tested whether the camera’s field of view (FOV) was sufficiently wide to spatially capture the range of fixations made by infants and adults during a naturalistic situation in the lab. For this purpose, we recorded infants’ and adults’ egocentric perspectives concurrently from a head-mounted eye-tracker (Positive Science Inc., USA [106]) and our EgoActive HMC. The eye-tracker measured the spatial location of fixations with high accuracy (2⁰ [106]) which allowed us to estimate whether fixated locations fell within our HMC’s FOV. We chose to integrate the EgoActive HMC with a head-mounted eye tracker because the latter allowed wearers to conduct naturalistic behaviors such as free play for infants or making tea for adults. Figure 11 illustrates the general approach.

The Positive Science head-mounted eye-tracker (the Tethered Laboratory Unit, Positive Science Inc., Rochester, NY, USA) consists of two cameras mounted on a headband: one which records the scene in front of the observer (located above the right eye; FOV: horizontal 81.80°, vertical 67.78°, diagonal 95.30°) and one which records the right eye (Figure 11). The scene camera captures a large proportion of the infants’ FOV [106]. In addition, we incorporated our EgoActive camera into the headset, aligned to the center of the forehead. Both our and the eye-tracking scene cameras were adjusted to have a field of view aligned with the infants’ line of sight.

Participants. For the purpose of validating the HMC for capturing what is likely to be fixated from the infant (6 and 12 months) and toddler (24 and 36 months) view, we recorded a period of free play between the infant/toddler and their caregivers (*N* = 32). For validating the HMC for adults (*N* = 9), we recorded a period of typical everyday behavior (i.e., making a cup of tea). Table 1 indicates the sample size included in the analysis for each age range and type of HMC.

**Procedure.** For the infant and toddler play, the caregiver was instructed to play with their child as they would normally do at home, using the toys and other objects present in the lab’s playroom. For the adult activity, the participants were invited to make a cup of tea in a dedicated area in the lab.

Given the dynamic changes in the visual environment in which the eye-tracking took place, an offline eye-tracking calibration procedure was adopted, as recommended for this type of situation [107]. This procedure was implemented in two stages. First, a series of pseudo-calibrations were conducted prior to the play for infants and prior to the tea-making activity for adults. They are similar to the typical online protocols (e.g., peek-a-boo game), and designed to allow the calibration of the eye-tracker for capturing fixations in different sections of the visual field, including variations in depth. For infants, all pseudo-calibrations were 5 points, while there was a combination of 9 (for fixations in depth) and 5 (for fixations in the lower visual field) points for adults. Second, after the recording session was completed, Positive Science Yarbus software (Positive Science, Inc., Rochester, NY, USA) was used to determine the point of gaze and superimpose the eye video with the scene video. This enabled offline calibration using the points from the pseudo-calibration as a reference.

**Figure 12:**
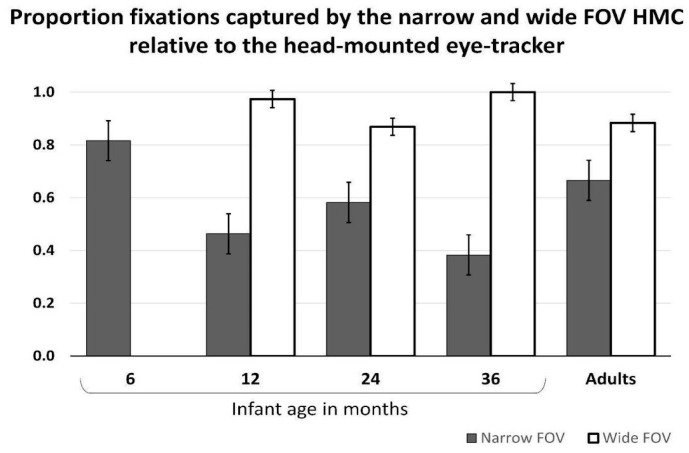
Proportion of fixations captured by the narrow and wide-FOV HMC relative to the head-mounted eye-tracker as a function of age. The error bars reflect the standard error of the mean.

Data processing and results. For the purpose of validating the HMC FOV, we extracted a 5 min segment from the recording session for each infant, toddlers and adult. The scene video superimposed with the point of gaze was temporally aligned with the HMC video. Custom-built Matlab (The MathWorks, Portola Valley, CA, USA) scripts were used to label each frame of the HMC video in terms of whether it contains the object that is visually fixated as indicated by the head-mounted eye tracker. We further calculated the proportion of frames from the total number of frames of the HMC video recording that captured the objects from the scene fixated by the infants, toddlers and adults. Overall, the narrow-FOV HMC captured a smaller proportion of the fixated spatial locations in the participants’ view (*M* = 0.60, *SD* = 0.33), relative to the wider FOV HMC (*M* = 0.93, *SD* = 0.10), and this was particularly the case for the older infants, toddlers, and adults (Figure 12). For the 6-month-old infants, the narrow-FOV HMC captured over 80% of the fixated spatial locations.

##### User Experience

The majority of the caregivers (97%) rated their experience with the HMCs as being positive, and 100% that it was easy to operate. Most caregivers also reported that they tended to forget that they were wearing the HMC (33% were not aware of its presence, 44% were somewhat aware of its presence). The majority of wearers reported that the HMC did not interfere with daily routines and activities (81%). Furthermore, the majority of the caregivers (67%) reported that the infant’s HMC was accidentally removed only infrequently. Taken together, these results indicate that the HMCs are comfortable enough that, at least based on the adults’ reports, the users tend to have little awareness of their presence, and that they are integrating fairly well with their daily routines and activities. This in part reflected by the fact that participants (infant and adult) tend to record with the HMCs for relatively long periods of time (*M* = 25 h 10 min/week, *SD* = 5 h 35 min, from the target of 28 h). Infants wore the HMC whilst this was recording for an average of 3 h 52 min/day (*SD* = 1 h) out of a total average recording of 3 h 58 min/day (*SD* = 1 h 1 min). During the period of time when the HMC was intended to be worn by infants for recording, it was removed on average 3 times (*SD* = 3 times) per day or not being worn for 6 min (*SD* = 7 min) on average per day. This indicates that the infants comply fairly well with the procedure for using the HMC. The fact that the infant HMC is only infrequently removed during use, also suggests that its physical features, including the temperature, are unlikely to cause discomfort.

#### 2.5.2. Validation Studies of EgoActive Body Sensor

We carried out three validation studies to evaluate different aspects of the EgoActive sensor, using complementary methodological approaches. First, we tested the reliability with which the EgoActive sensorrecords ECG relative to a commercially available wearable sensor during naturalistic social interactions. Second, we assessed the effectiveness of the EgoActive sensorin accurately recording ECG and acceleration for long periods of time, in line with its intended use in the home environment, relative to an ECG simulator. Third, we used subjective reports from the caregiver to assess whether infants and caregivers had a positive experience wearing and operating the sensor during their daily routines and activities.

##### Comparison with Commercially Available Wearable Sensors—Short Recordings

This study examined the EgoActive sensor’s capability to record ECG and acceleration data during social interactions in a naturalistic setting. Caregivers and infants were invited to play as they would do at home for 20 min (range 14–24 min) in the lab playroom. Participants were able to play with any toy or objects they wished from those available in the playroom. 

We compared our EgoActive sensor to the commercially available Biosignalsplux wearable sensor (PLUX Biosignals, Portugal). Our interest was in testing the device on infants since their developing motor skills present the risk of a wider range of noise that can occur within the ECG. In this study, we focused on a short period of play in the lab rather than longer durations in the home environment, predominantly due to the characteristics of the Biosignalsplux wearable sensor (PLUX Biosignals, Portugal). It is fairly bulky for an infant, and involves several wires that connect the electrodes to the hub. Although the Biosignalsplux sensor (PLUX Biosignals, Portugal) has some limitations as a wearable, it stands out for its high sampling rate (up to 1000 Hz) and capability to integrate multiple sensors. As a result, it is a good resource for assessing real-life situations and gathering valuable information. Additionally, in our experience, a free play period in the lab playroom exposes several situations that can impact ECG signal quality, similar to the home environment. A sampling rate of 500 Hz was chosen for the Biosignalsplux (PLUX Biosignals, Portugal) wearable sensor in order to provide a high-resolution heart rate to allow for accurate alignment with the EgoActive sensor.

**Participants.** The sample included 3-, 6-, 12-month-old infants and 24- and 36-month-old toddlers (Total *N* = 30; Table 2).

**Procedure.** Before attaching the Biosignalsplux wearable sensor (PLUX Biosignals, Portugal) and EgoActive sensors to the infant’s chest, they were synchronized using a light signal from the base unit. The Biosignalsplux wearable sensor (PLUX Biosignals, Portugal) was placed on the left side of the chest, while our EgoActive sensor was placed on the right side. This configuration allowed both devices to measure the same underlying source for ECG and heart rate. The heart rate was extracted from both devices, aligned with the Biosignalsplux sensor (PLUX Biosignals, Portugal), and then the difference between the two signals was calculated at each time point for the Biosignalsplux signal. It is worth noting that while some minor differences in noise level and ECG morphology arise from the different sensor locations, the biomechanics underlying the pumping heart arise from the same source for both locations. These similar biomechanics lead to only small expected discrepancies between the two heart rates (as shown in [108], where 2 ms was the maximum standard deviation of R–R intervals calculated between different measurement locations). The overall quality of the match between heart rates was measured by the proportion of the heart rates that fell in agreement within 5 bpm, as defined by Equation (1). The 5 bpm were chosen as similar to the mean absolute error between wearable devices and ambulatory ECG recordings over a 24 h period [109], and was a way to exclude incorrect R-peak detections in a dynamic recording setting, while still preserving those R peaks that matchup between the two devices, yet allowing for some minor deviations between the two.

(1) $$Proportion\;signal\;agreement=\frac{\#\;HR_{Plux}\;beats\;within\;5bpm\;of\;HR_{EgoActive}\;beats}{Total\;\#\;HR_{Plux}\;beats}$$

**Data processing and results.** We obtained concurrent ECG recordings from 30 participants (*M*_OverlapLength_ = 27 min, *Min*_OverlapLength_ = 15 min; *Max*_OverlapLength_ = 43 min). The ECG signal from the two devices were aligned via a two-parameter optimization: shift and stretch of the ECG signal from the EgoActive sensor. The stretch parameter corresponds to the relative clock speed (see Section 2.5.2, Comparison with ECG Simulator—Long Recordings), while the shift parameter primarily captures the time offset between the two devices being turned on.

**Figure 13:**
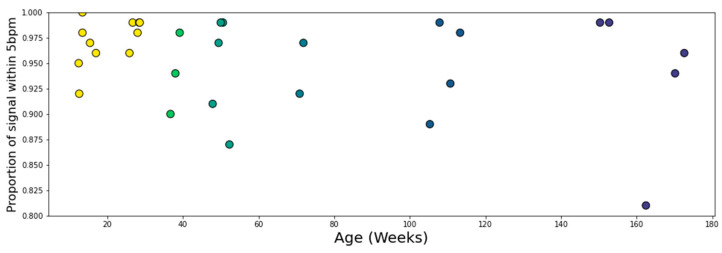
Proportion of signal agreement (within 5 bpm) between the Biosignalsplux^TM^ and EgoActive wearable sensors as a function of age (in weeks). Each dot represents data from a participant. The color-coding of the dots corresponds with age in order to allow cross-reference with Figure 14 and Figure 15.

The average signal agreement within 5 bpm was 95.4% (*Min* = 81%; *Max* = 100%), with 26/30 sharing more than 90% of the signal and 19/30 sharing over 95% of the signal. There was no statistically significant correlation with age (*r* = −0.24, *p* = 0.21), suggesting that the age of the participant was not a factor in signal quality (Figure 13).

**Figure 14:**
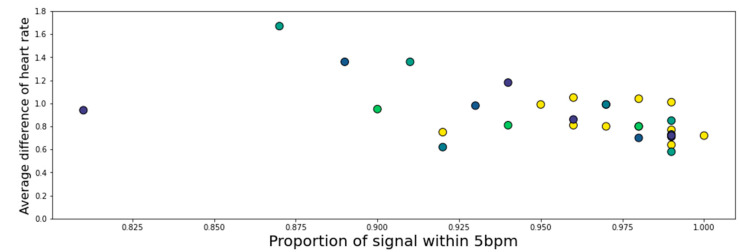
Relationship between average difference of signal agreement (measured by the devices within 5 bpm) and corresponding average absolute difference in heart rate. Each dot represents data from a participant. The color of the dot represents the participant’s age (in weeks), with darker colors representing older participants (see Figure 13 and Figure 15).

In order to justify the 5 bpm limit and also to quantify the combined effect of the reduced sampling rate of the EgoActive sensor and the differing sensor locations, the average signal within 5 bpm was compared against the proportion of total signal within 5 bpm. The mean absolute difference for those regions within 5 bpm was calculated for each participant, and is shown in Figure 14. The mean of these measurements was 0.906 bpm (*Min* = 0.58; *Max* = 1.67 bpm), and 23/30 of the recordings had a mean difference < 1 bpm for these regions. These measurements displayed a significant negative correlation (*r* = −0.55, *p* = 0.002), with noisier signals displaying less agreement within 5 bpm and higher average deviations within 5 bpm. However, removal of the five signals with the lowest proportion of matching signals reveals that the identified negative linear association is not present in the remaining twenty-five signals (*r* = −0.26, *p* = 0.21, *M* = 0.836 bpm). This serves as a rough estimate for the difference in detected heart rate due to differences in device location and sampling rate.

**Figure 15:**
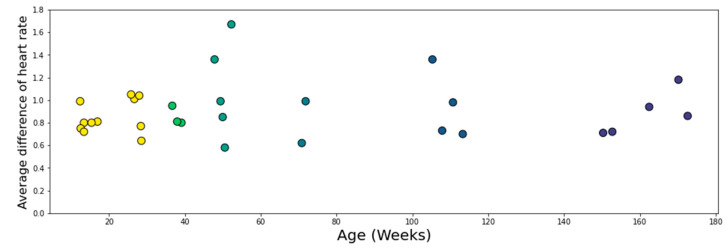
Relationship between average difference of signal in agreement (measured by the devices within 5 bpm) and age (in weeks). Each dot represents data from an infant or toddler. The color-coding of the dots corresponds with age in order to allow cross-reference with Figure 13 and Figure 14.

Figure 15 shows that there was no correlation between the average difference in heart rate (for areas < 5 bpm difference) and age for the participants studied (*r* = 0.045, *p* = 0.81). This shows that the EgoActive device has the same level of effectiveness relative to the Plux device across the ages measured. 

##### Comparison with ECG Simulator—Long Recordings

A set of validations evaluating the effectiveness of long-term EgoActive sensor recordings was also carried out. The sensor is designed for long naturalistic recordings, but relies on an internal clock for time keeping. As such, it is important to assess the accuracy of this clock, and whether any drift occurs over time. Furthermore, while the comparison with the BiosignalsPlux device is important as detailed above, it is limited by the absence of the ground truth for both devices. For example, both the BiosignalsPlux (PLUX Biosignals, Portugal) and EgoActive sensors could be subject to incorrect recordings from high motion and other sources of infant noise. In order to address this limitation and investigate the performance of our sensor over long periods of time, a simulator, TechPatient Cardio Version 4 (HE Instruments LLC, Lake Worth, FL, USA), was used.

**Procedure.** The simulator was set to generate a uniform 150 bpm ECG signal, and five recordings between 2 and 10 h were carried out (*M* = 4.5 h, *SD* = 3.1 h) on five separate EgoActive sensors. 

**Data processing and results.** The accuracy of the sensor was then tested by dividing the expected mean time gap between ECG peaks (0.4 s) by the recorded mean time gap between ECG peaks for every half hour. Unlike the naturalistic signal in the previous study, the ECG simulator is essentially not noisy and provides the ground truth for comparison purposes. As stated in Section 2.3.2, the sensor writes data to a .dat file every 5 min. This process took no longer than 0.08 s on any recordings (a duration long enough to miss the ECG peak) and the only missed peaks occurred during the data transfer process, i.e., while converting to .dat files. All other ECG peaks were fully detected and as such, no further signal quality analysis will occur here. 

**Figure 16:**
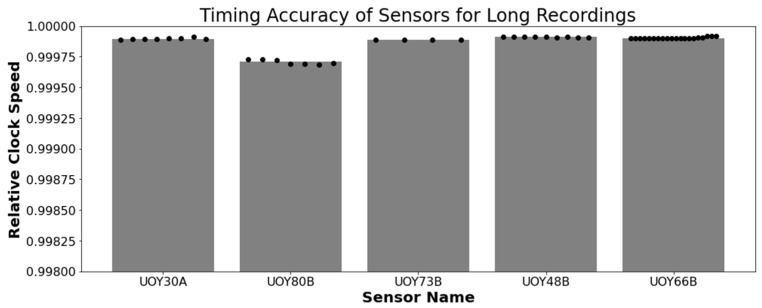
The average relative clock speed is shown as the height of the bar charts, and the relative clock speed for each half hour is shown as a scatter plot for each sensor. The slight increase for UOY66B occurs after 8 h of recording.

The calculated instantaneous heart rate was batched into averages for each half hour. This provides an accurate representation of the internal clock during that time. Any significant change in half-hour-averages will highlight the consistency of the recording (i.e., variation and drift of the clock). Figure 16 shows both the average relative clock speed, and the distribution of clock speeds across the recordings. The overall average relative clock speed was around 0.9999, i.e., the clock would be 0.1 s ahead of a real-time clock after 1000 s of recording, or alternatively the device would record a 150 bpm heart rate as 149.985 bpm. We therefore apply a 0.9999 correction to any output time series in order to minimize inaccuracies further. There are some minor variations within a recording, but no clear trend of drift for the clock speeding up or slowing down for a given sensor. Three recordings trended down, two trended up, and the average absolute drift was only a shift of 2.3 × 10^−6^ in relative clock speed per half hour.

##### User Experience

Similar to users’ experience with the HMC, a very high percentage of users rated their experience with the EgoActive sensor as positive (89%) and easy to place on both adults (100%) and children (78%) despite their inexperience with the devices and the electrodes. Parents reported that it was fairly easy to remove the sensing pads and body sensor from their body (78%) and their child’s body (56%), and that wearing the body sensor did not interfere with their or their child’s daily activities (100%). In fact, participants (infants and adults) tend to frequently record with the body sensor for long periods of time (*M* = 23 h 58 min/week). We identified ECG signals on infants wearing the body sensor for *M* = 3 h 52 min/day (*SD* = 1.18). These results indicate that the body sensor is a wearable device that can comfortably be worn for extended periods of time by adults and infants as young as 6 months.

## 3. Software

We now describe the software developed for the EgoActive platform. This falls into two categories. First, we have developed an Android app in Java (described in Section 3.1) which runs on the base unit tablet and which provides synchronization and data management functionality. This app is used by participants while the platform is deployed during data collection. Second, we have developed a number of software tools in Python for automatic preprocessing of the raw data captured by our devices prior to further extraction of meaningful features. This is run after a round of data collection is complete on dedicated processing servers. Specifically, these preprocessing tools must: 1. extract the synchronization codes from data recorded by each device so that we can match and temporally align the different modalities of data (Section 3.2.1); 2. extract heart rate from the raw ECG signal (Section 3.2.2); 3. preprocess the accelerometer data into a usable form (Section 3.2.3); and 4. automatically detect which portions of the HMC video data are of sufficient quality to be usable (Section 3.2.4).

### 3.1. Android App for Device Synchronization and Data Backup

**Figure 17:**
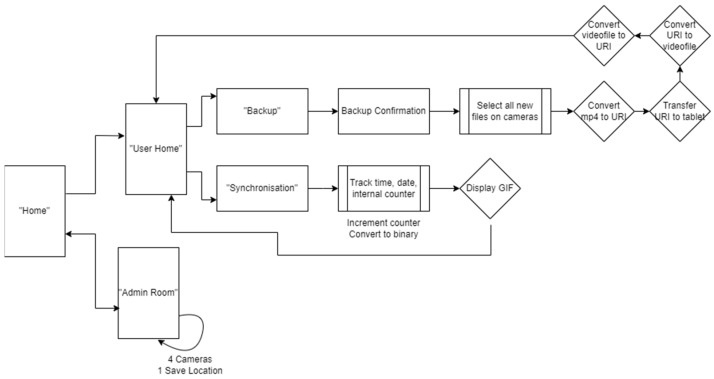
Data flow diagram of the EgoActive App showcasing the layout and navigation of the Android application.

The Android application was developed to enable the synchronization of the devices through the display of a 10 bit binary pulse at the beginning of a recording session; and in order to backup the data from the HMCs and sensors onto the Android tablet. The application’s overall layout is depicted in Figure 17. The “Admin Room” is completely isolated from the rest of the application and inaccessible from the “User Home” area. Moreover, the “Admin Room” is safeguarded by a password, making it unlikely for unauthorized access. Inside the “Admin Room,” there is a section dedicated to allocating camera and sensor URIs, effectively designating them as “Safe foreign devices” for data transfer. Only devices handled by the specific researchers assigned to each box are marked as safe. Appendix B includes a detailed description of a single run through the app. 

The software was developed for and tested on the Samsung A8 with Android 11 that we use in our base unit. In principle, the application could work on other devices or versions but this has not been tested. The version of Android is important due to its security implementations and stopgaps. Android 11 utilizes Uniform Resource Identifiers (URI) as the internal file handler of choice. URIs exist to securely move files between internal and external folders, in order to deal with the critique of previous Android version debug permissions (ADB) [110].

**Figure 18:**
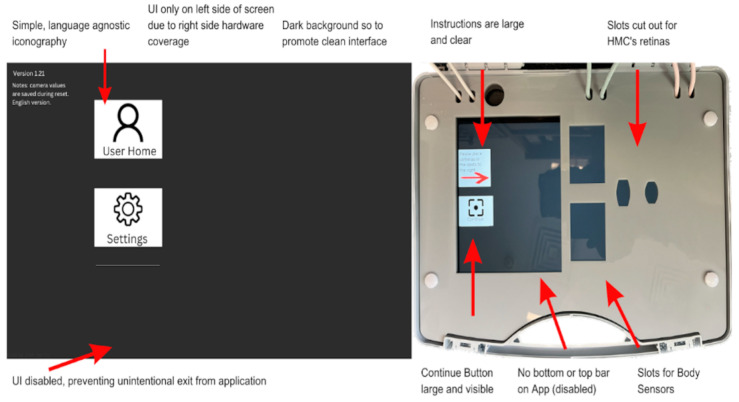
An annotated sample image from the application and an example of how the application works in combination with the hardware modifications to make use cases clear.

We also modified the tablet settings to prevent users from leaving the app and accessing the home screen. This mitigates the risk of accidental app closure or unintended user actions through the use of the ‘home bar.’ This ensures the stability and reliability of the application while also ensuring users do not get lost while using it. Figure 18 shows how a combination of the UI of the app and the modifications made to the hardware encourages users to interact with the application as we intended.

We now describe the design and implementation of the two key features of the application: synchronization and data backup.

#### 3.1.1. Design of the Synchronization Signal

The synchronization signal serves three purposes. First, it enables matching between recording sessions from different devices using the synchronization bitcode. Second, once correspondence between sessions has been established, it enables precise temporal synchronization between time series data captured by all devices. Third, since the body sensor is not equipped with a RTC, by encoding date and time information into the synchronization code we are able to augment body sensor recordings with this information. For the first purpose, we require that the generated synchronization codes are sufficiently distinct that, when combined with the additional information from a camera’s RTC and the ordering of recorded sessions on the body sensor, allow unambiguous matching between sessions. This must include robustness to user error so that we can detect and (if possible) recover from scenarios such as following the synchronization procedure for only a subset of the devices or running the synchronization process multiple times in quick succession. 

For usability, we wish to minimize the time required to record the synchronization signal. However, we are limited by the effective sample rate of the camera. While the camera averages 30 Hz, this can drop as low as 20 Hz during normal performance. This is further compounded by the rolling shutter capture, automatic exposure and white balance adjustment of the camera. These add noise to the signal and make it non-trivial to later decode to a binary signal. For this reason, we limited the synchronization signal to 5 Hz which we found could be reliably decoded from video data in practice.

**Figure 19:**
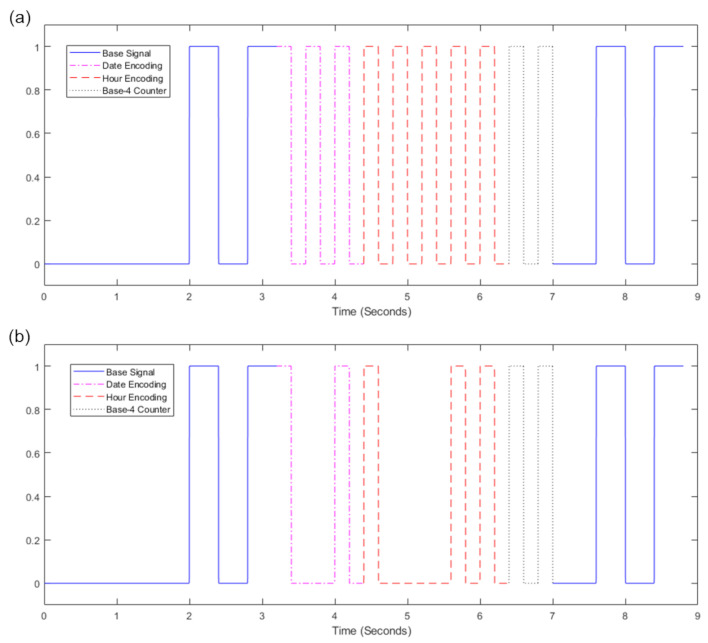
Examples of the 10 bit synchronization signal that generates a unique code to temporally align different devices. The solid blue line represents the constant base sequence used to locate the synchronization signal. The dashed colored lines represent the synchronization signal that encodes the date (magenta), hour (red) and counter (grey). (**a**) The unique code is: 1111111111 (all 10 bits “on”). (**b**) The unique code is: 1011001111.

The synchronization signal consists of a sequence of 10 temporally consecutive 400 ms periods generated by the EgoActive App, representing a 10 bit code (see Figure 19). The value of each bit is represented by the intensity within that period. An “on” bit is represented by a 200 ms high-intensity pulse, followed by a 200 ms low-intensity period. An “off” bit is represented by a 400 ms low-intensity period. The first 3 bits encode the synchronization date (0–7, specifically the day of the month modulo 8). The next 5 bits encode the synchronization hour (0–23). The final 2 bits are a counter that encode the number of synchronizations initiated within a single hour. This counter disambiguates situations in which users synchronized devices more than once within a single hour (up to a maximum of 4). Thus, the tablet date and time, and counter can generate a unique synchronization code across all devices for a recording session. In its current implementation, unique synchronization codes are generated if the total recording period is 8-days or less and users do not synchronize more than 4 times within a given hour.

To facilitate locating the 10 bit code recorded by the HMC or sensor, we generated a base sequence before and after the synchronization signal. The beginning of the synchronization signal was preceded by a 2000 ms low-intensity period, followed by a high-intensity pulse, low-intensity period, high-intensity pulse sequence (400 ms each). The end of the synchronization signal was then followed by a low/high/low/high-intensity sequence (400 ms each; see solid blue line in Figure 19). Finally, a full green screen was presented for 1000 ms. This indicated to users that the synchronization process was completed. The green screen further facilitated locating the 10 bit code in videos (see Section 3.2.1).

We playback the synchronization pattern on the tablet screen by setting the screen color to black (R = G = B = 0) for 0, white (R = G = B = 255) for 1 and green (R = B = 0, G = 255) for the post-signal period. In practice, to avoid having to generate the synchronization patterns on-the-fly (and therefore relying on the timing of the device), instead we precompute all 2^10^ = 1024 synchronization codes and save them as animated GIF format images. When the synchronization process is initiated by the user, the date and time of the tablet clock along with its internal 2 bit counter is used to select which GIF should be retrieved and presented. Since GIF display is not natively supported on Androids, we use the MavenCentral Android GIF viewer [111].

#### 3.1.2. Data Backup

The application also has the function to backup the HMC recordings onto the Android tablet. When the HMCs are connected to the base unit via their charging cables, the backup function within the app can be initiated. This automatically transfers all new video files (i.e., those that have not yet been backed up) to the internal SD card in the tablet via a URI transfer. This provides redundancy in case the HMC SD cards are subsequently damaged but also provides some initial structuring of the data: video files from all four cameras are now stored in a single location with separate folders for each camera, date and hour. By leveraging the built-in security measures of an Android device, the application provides a safe and reliable backup solution. At the end of a data collection phase when the base unit is returned to the researchers, the backup process is run a final time to ensure all camera files have been copied to the tablet. Then, the researchers need only copy data from the tablet SD card (and body sensor SD cards) to a central file store.

To implement the data transfer process itself, we use SimpleStorage API [112], an open-source API responsible for representing the files as URIs for the purpose of mobility between devices. SimpleStorage is simple and secure, and the fact that the API is open-source means safety can be verified through analysis of the code. Each file is associated with a specific Multipurpose Internet Mail Extension (MIME) type. In the Android operating system, MIME types are further categorized into ten subtypes known as URIs. For our application, this means that only files conforming to the correct MIME/URI structure are transferred during the backup process. In our case, video files transmitted by the designated cameras within the password-protected “Admin Room”.

### 3.2. Software for Preprocessing Raw Data

In the current implementation of the EgoActive platform, data from the HMC and sensors are stored as 5 min data files (MP4s for HMC videos, and custom binary files for sensor data). Thus, as part of the platform, we provide a set of software tools implemented in Python to help users preprocess the raw data files that can facilitate analyses of the wealth of multimodal data. In particular, we describe our tools for extracting the synchronization signal from devices, extracting heart rate from ECG, accelerometer preprocessing and HMC video quality labelling to identify potentially unusable segments.

#### 3.2.1. Software for Temporal Synchronization of HMC and Body Sensor Data

We aim to build a fast and computationally efficient solution to accurately locate and decode the 10 bit synchronization signal recorded by the HMCs and body sensors. This decoding generates a unique code that can be used to temporally align different devices. 

The same algorithm is used to locate and decode the synchronization signal in both data modalities; however, there are some minor differences in preprocessing video versus the luminosity signal recorded by the body sensor. These are described first.

**Body sensor luminosity signal preprocessing.** The body sensor stores the recorded luminosity as a binary signal sampled at approximately 250 Hz. This signal is not uniformly sampled in time and so we uniformly resample at 500 Hz using nearest neighbor interpolation to maintain a binary signal.

**Figure 20:**
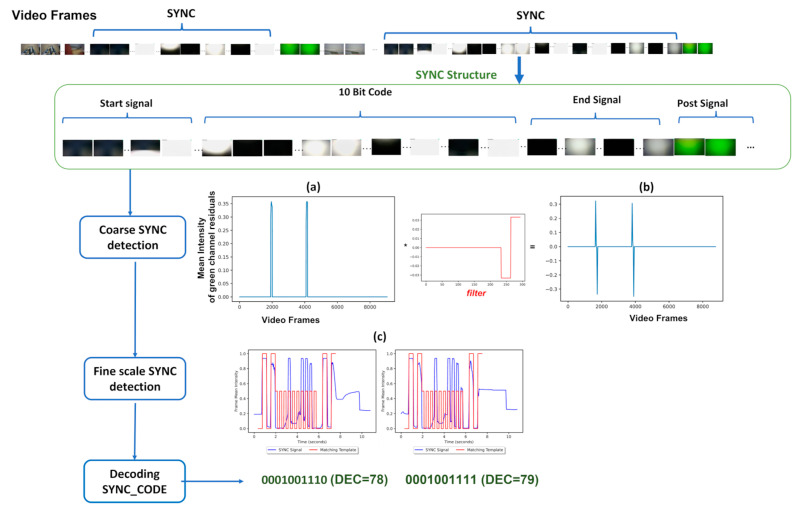
An overview of the synchronization signal detection and decoding process for video data. The initial raw video frame data (**top**) is converted to a 1D greenness signal (**a**) which is used to coarsely locate candidate segments by convolution with a filter providing a response (**b**) which is maximal when the filter detects the transition to the post-signal green frame. Precise location is found by sliding a template signal ((**c**)—red) over the mean intensity signal ((**c**)—blue) and computing the normalized cross-correlation (position of maximum response shown for two detected segments). Finally, the 10 bit code is extracted by thresholding the mean of the signal within the “high” periods of the 10 bit signal (**bottom**). The “*” is the convolution operator.

**Video preprocessing.** The video data are also non-uniformly sampled in time (the video encoder in our HMC uses variable frame rate encoding). First, we uniformly resample the videos to 30 Hz using nearest frame interpolation and downsample resolution by a factor of 0.5 to 960 × 540 pixels. Since video data are written in 5 min blocks, we also identify consecutive video files and stitch them into a single continuous file. This resampling and stitching is done efficiently using FFmpeg [113] running on a GPU. The resampled and stitched videos are saved for later processing. To extract the synchronization signal from these videos, we convert to a 1D time series representing the mean intensity. In order to perform this process efficiently, we implement this as spatial global average pooling in PyTorch (The Linux Foundation, San Francisco, CA, USA) and perform the processing on the GPU operating on batches of frames in parallel. This provides a signal for each color channel. From this, we extract two signals:

1. The ‘greenness’ signal is defined as max(0,G-R-B). This signal (see Figure 20a) has a large value when the mean color of the frame is green, i.e., when the average value over pixels is significantly larger in the green channel than red and blue, otherwise it is zero.
2. The average intensity signal is simply the average over the three color channels (see blue curves in Figure 20c).

The average intensity signal from video frames is continuous (not binary) and significantly noisier than the luminosity signal from the body sensor. For this reason, for the videos we run an initial coarse search to locate candidate segments within the video that may contain a synchronization signal. This exploits the additional information of the green screen presented at the end of the synchronization signal. To do so, we simply search for points of transition from zero greenness to high greenness. This is implemented efficiently as a PyTorch 1D (The Linux Foundation, San Francisco, CA, USA) convolution layer with a single fixed filter (shown in Figure 20a in red) and executed on the GPU. This gives a large response (see Figure 20b) when the transition from the −1 segment to +1 segment aligns with the increase in greenness. The filter is zero padded at the front such that the position of the filter when encountering a peak response provides a frame index that occurs before the start of the synchronization signal. Finally, we run a peak finding algorithm on this signal to provide the candidate segments to search for synchronization signals. We constrain the peak finding such that the minimum distance between peaks is 500 frames (16.7 s). This time was determined as the minimum required for a user to run two consecutive synchronization processes in the app and avoid detecting the same synchronization signal twice if there is noise in the greenness signal.

**Synchronization signal location.** After preprocessing, both the body sensor luminosity and video data provide 1D, uniformly sampled time series. We now search these two time series for the precise location of any synchronization signals. Exactly the same algorithm is used in both cases, the only difference being that, for the videos, we only search the already-identified candidate segments whereas for the body sensor luminosity we search the entire sequence. For the video signals we also upsample to 100 Hz using linear interpolation. To locate synchronization signals, we slide a template signal over the raw signal and compute the normalized cross-correlation (NCC). Since the 10 bit code is unknown, we do not know whether the actual signal contains zeros or ones in this portion of the signal. Hence, in the template, we set the possible high parts of the 10 bit code signal to a half value (see Figure 20c, red curve). This can be seen as the average of all possible 10 bit codes and therefore the best choice for searching as the ‘expected’ signal. The location of synchronization signals is given as the local maxima of the NCC response. We record the position of these detections to use later for temporal alignment. We apply an additional threshold to the NCC maxima and only retain those whose NCC value is above the threshold. This removes spurious detections caused by noise in the signal. The threshold is set conservatively enough to deal with 0.4 s of incorrectly transmitted synchronization signal. For the body sensor, this is wide enough to deal with a signal which was recorded during the data transfer (the conversion of data to a .dat file, which occurs every 5 min), the main source of corruption that could potentially occur during the process for the body sensor. 

We validated the synchronization signal detection algorithm on a dataset of 1444 time series, of which 1152 contained at least one synchronization signal for a total of 1218 synchronization signals in total. All signals were successfully detected and only three false detections occurred in all the time series analyzed. This implies that the synchronization signal is sufficiently structured to avoid being activated by random noise, while the threshold is also low enough to ensure all genuine signals are detected.

**Synchronization signal decoding.** Having located synchronization signals in the body sensor luminosity or video time series, we extract the 10 bit code by finding the mean of the recorded signal over each part of the signal that could contain a “high” signal. Specifically, we decode the *i*th bit through Equation (2).

(2) $$b_{i}=\frac{1}{e_{i}-s_{i}}\overset{e_{i}}{\underset{j=s_{i}}{\sum }}x_{j}>t$$

*s_i_* and *e_i_* are the start and end positions of the high segment of the *i*th bit, *x* is the measured signal and *t* is a threshold. For the (binary) luminosity signal, we use a threshold of 0.5 and in practice implement this by taking the median value over the segment. For the (continuous) video signal, we use a threshold of 0.3. This lower value is required since a white screen is usually recorded as an intensity less than 1.

**Synchronization signal matching.** Having extracted synchronization codes from body sensor and video data, we are able to find matches between different time series both within and between the different modalities of data. The 10 bit binary code can be converted to a decimal representation for easier matching. Once matches are identified, the devices are temporally aligned by setting *time* = 0 to the start of the synchronization signal (i.e., start of bit 1 in the 10 bit code; Figure 20). Note that synchronization signals might be recorded by some but not all of the devices. Hence, we cannot guarantee a one-to-one match between all detected synchronization signals. Synchronization codes and hence matches should be unique in all but pathological cases (where >4 user-initiated synchronizations are run within a single hour). In these rare cases, we can still disambiguate the matches. Recordings on all devices are ordered by time and the sequence of matched detections must preserve this temporal ordering. In addition, since the HMCs are equipped with real-time clocks, date/time information in body sensor synchronization codes can be used to select HMC recordings at a similar time. Having established a match and with the corresponding locations of the signal within the original time series, we can define all time series with respect to the reference synchronization time providing the final synchronized time series data. Where a time series contains multiple synchronization signals, we use the latest one as the start of the time series.

#### 3.2.2. Body Sensor ECG Processing

**Figure 21:**
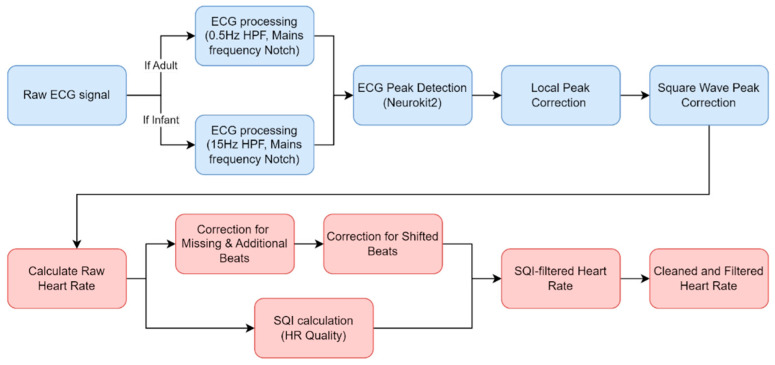
Proposed pipeline for processing recorded raw ECG signal into usable heart rate (HR) signal. ECG processing steps in top row (blue); HR processing steps in bottom row (red).

The raw ECG signal captured by the EgoActive sensor is processed in two stages, each encompassing several steps: first the raw ECG is processed into a heart rate (HR) signal (the main steps are detailed algorithmically in the top row blue boxes of Figure 21); second, the HR undergoes a process of cleaning in order to yield reliable HR signal stretches to be used in subsequent analyses, along with noise and artefact identification for unreliable HR signal stretches (the main steps appear in the bottom row red boxes of Figure 21).

**ECG processing and R-peak detection.** In an ECG, a detection algorithm seeks to identify the QRS complex, a characteristic feature of the ECG with an R-peak in the center [114]. The set of detected R peaks is then converted into the instantaneous heart rate (calculated in beats per minute) by using the formula in Equation (3) below. Here, t_peaks_ refers to the set of time indices (in seconds) corresponding to the labeled R peaks and Δ denotes the differencing operator which calculates the inter-peak gaps.

(3) $$HR\left(bpm\right)=\frac{60}{\Delta t_{peaks}}$$

Initial preprocessing is required to filter noise out and to allow for the QRS complex detection [115], the source of characteristic R peaks within the ECG. With adult ECG, a high-pass filter of 0.5 Hz is used in combination with a notch filter at the mains-electricity frequency (e.g., 50 Hz or 60 Hz). The 0.5 Hz threshold along with a notch filter is the default setting in the Neurokit2 open source Python (Python Software Foundation, Wilmington, DE, USA) library for ECG peak detection [116] and in other studies (e.g., [117]), while also being the same lower bound used for monitor-quality ECGs [118]. The Neurokit2 peak detection method (default settings) was used to detect R peaks on the preprocessed ECG signal [116], where the QRS complexes are identified based on the steepness of the absolute gradient of the ECG, and the local maxima are identified as the R waves.

**Figure 22:**
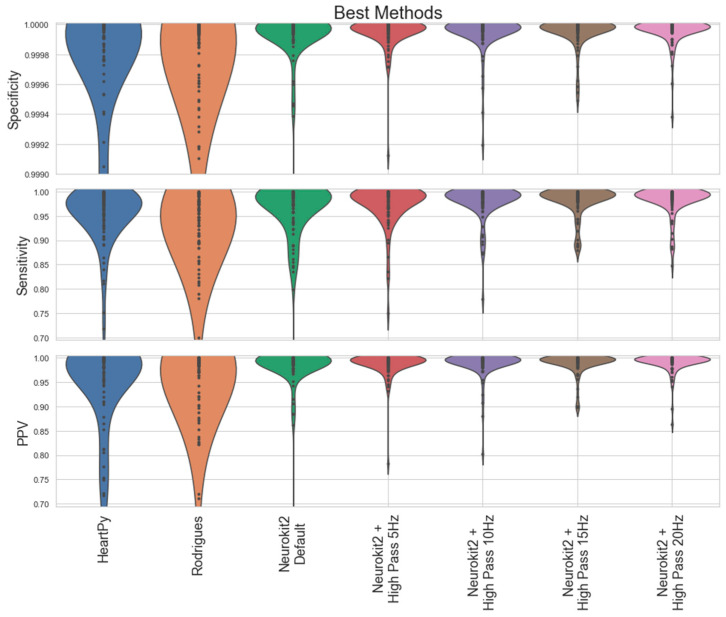
A demonstration of the improved performance of the 15 Hz HPF compared to other HPF options and three pre-existing methods: the HeartPy package approach [121], Rodrigues et al. [122] and the Neurokit default pipeline. These comparisons all include the local novel correction. The specificity (true negative rate) measures successful non-detection of incorrect peaks, the sensitivity (true positive rate) measures successful detection of correct peaks, and the positive predictive value (precision) measures the proportion of correct peaks detected to all peaks detected for a given method.

Infants have a higher heart rate than adults [119] and as such have a different frequency content within the signal. In comparing bandpass filtering ranges for preprocessing of infant ECG, a 1–17 Hz bandpass was found to approximately halve the R-wave peak compared to the 0.05–150 Hz option [120]. In order to preserve the R-wave peak in children, we experimentally verified the optimal set of filters on a hand-labeled dataset of infant ECGs (*N* = 88, age range: 5–42 months, mean duration: 33 min). A 15 Hz high-pass filter (HPF) with a notch filter at the mains-electricity frequency was found to be the most effective approach, and so was adopted (Figure 22). 

The specificity (true negative rate), sensitivity (true positive rate), and positive predictive values (precision) were used as measures of success. While the ability to detect peaks (sensitivity) is highly valued, it is arguably more important to not predict peaks where none exist (specificity, positive predictive value) as it is easier to recover small amounts of lost signal than to reject incorrectly labeled beats. Fortunately, the 15 Hz HPF + Neurokit2 approach was the best across all metrics, and so no tradeoff had to be made.

A novel local correction relative to the unfiltered signal was then carried out in order to counteract the shifting peaks effects of frequency filtering. This correction iteratively searches for the largest peak ±0.01 s either side of the peak location on the processed ECG, to check for a larger local peak within the raw unprocessed ECG.

**Figure 23:**
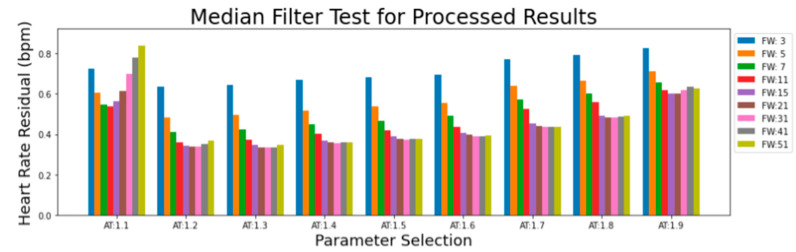
Graph showing the choice of optimal parameters for detection of missing and/or additional beats in the heart rate. FW is the width of the median filter, AT is the multiplicative factor to define the acceptance threshold.

**Heart rate processing.** Missing R peaks translate into artificially low heart rates. If the heartbeat was uniform, the heart rate due to the missing peak would register as precisely half the value of the neighboring heart rates. However, since heart rates exhibit constant variation, a more sophisticated algorithm is needed to automatically detect missing R peaks in a ground truth. Conversely, an additionally detected peak would inflate the heart rate artificially. Beat-to-beat comparisons are reported in the literature to identify mislabeled R waves, e.g., setting thresholds for detecting longer or shorter times between subsequent R waves as missing or incorrect R waves, respectively [123]. As the real-time necessity for beat-to-beat comparisons ceases for post-processing, our approach is to apply a threshold over a wider beat-window in order to robustly account for noise, hence missing/additional R peaks were found by significant deviations from a local median. The optimal parameters were chosen to minimize the average residual heart rate between the processed heart rate and a labeled ground truth in an infant dataset (*N* = 88) (see Figure 23, where a filter width of 31 and acceptance threshold of 1.3 led to the lowest average heart rate residual of 0.334 bpm). Any R peaks that fall outside that region are removed and the gap is filled by linear interpolation from the nearest R peaks that fall within the local median range.

**Figure 24:**
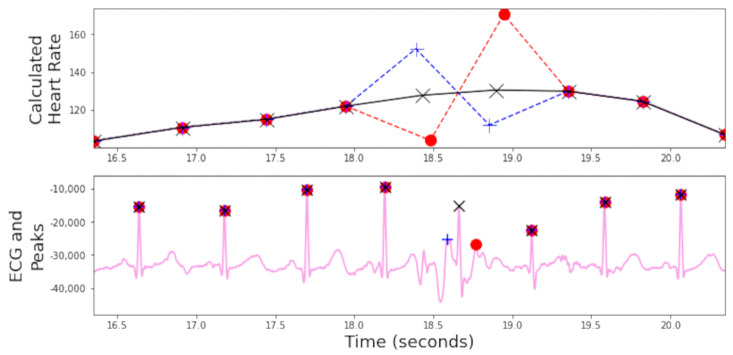
The bottom figure shows the raw ECG (magenta line), the top figure shows the derived heart rate using Equation (3). The effect of an early (blue line and blue +) or late (red line and red circle) label on the heart rate when compared to a typical beat (black line and black ×).

Wrongly located R peaks can still be undetected by the median filter approach. A useful observation is that an early-labeled beat will lead to a much greater heart rate rise and then a much steeper heart rate drop than would typically appear within a natural signal (Figure 24). A late-labeled beat will do the opposite. This motivates our proposal to search for the alignment of three consecutive sign changes concurrently with a large variation in the heart rate difference (>15 bpm for the first and third heart rate gaps, >25 bpm for the middle gap), thus identifying the mislabeled beats within a signal, provided the neighboring beats are correct.

Methods for assessing HR quality reported in the literature include checking for non-stationary signal, viable heart-rate range, and high signal-to-noise ratio (SNR) [124], or extracting shapes and behaviors of the signal and grouping the samples by an agglomerative clustering approach [125]. Kramer requires the user to accept/reject the signal in full, an approach which is not efficient for long naturalistic recordings. Rodrigues’s approach required periods of noise and signal, and became computationally inefficient for longer recordings. For the newly developed sensor, a custom algorithm was designed to detect areas of good quality heart rate signal within long recordings in a computationally efficient manner.

The beat correction algorithm for missed/additional beats was used as an initial measure of signal quality. Figure 24 highlights that correctly labeled R peaks will typically fall inside the expected bounds, whereas incorrectly labeled R peaks are likely going to either cause a steep increase or decrease in heart rate (for additional or missing labels, respectively). By calculating the proportion of “wrong” labels within a given filter width, a rolling measure of heart rate signal quality is calculated, hereby referred to as the “signal quality index” (SQI). An SQI vector was created and used to identify signal locations where less than 75% of the recorded beats within a sliding window width of 31 beats deviate by more than 1.3 from the local median. This vector works as an indicator of unreliable regions (i.e., the local median indicates the existence of poor signal within the measured region). In order to make the indicator more specific, various manipulations were used. The regions of good SQI were then grown according to whether specific beats at the boundary fell inside the local median range. Conversely, any continuous regions >3.5 s long of recordings outside the local median were set to zero SQI, as were any gaps in heart rate longer than 2.5 s. Additionally, any good regions <5 s long were set to zero SQI in order to only leave segments of a reasonable size. These parameters could be tailored depending on how long of a heart rate region is useful for a given academic question. 

#### 3.2.3. Acceleration Processing

The three calibrated orthogonal axes signals from the use of the body sensor accelerometer are presented in gravitational units (g). Data were collected for the 29 infants and toddlers aged between 3 and 36 months (14 ± 10 months). Periods of no signal, and the 5 points before and after, were identified and removed as <0.001 g from a 5-point moving standard deviation. Further, the bottom and top 0.05% of data points were removed as potential outliers. The absolute peak acceleration across all three axes was 1.56 ± 0.24 g. Simultaneous data were obtained using the Plux ACC accelerometer (Biosignalsplux, Portugal), and provided good agreement with 1.88 ± 0.49 g. With the EgoActive device, for three participants there were occasional peak values exceeding the accelerometer limits of ±2 g. As absolute values above 2 g were infrequent, an accelerometer range of ±2 g is suitable for 6–36 month old infants in a free-moving environment when the device is located on the left lateral superior chest that will dampen body accelerations through its position proximal on soft tissue away from skeletal bony landmarks. Combining the three axes provided resultant accelerations of 2.03 ± 0.40 g (EgoActive) and 2.47 ± 0.55 g (Biosignalsplux—Biosignalsplux, Portugal). These peak resultant accelerations during a free-moving environment are greater than those found when supine without toys, under a play gym or in a car seat of approximately 0.5 ± 0.2 g found for infants aged 5.2 ± 2.3 months with the accelerometer placed on the ankle [126]. 

#### 3.2.4. HMC Quality Control Processing

Since we rely on non-expert users to operate the devices and since data are captured in uncontrolled, natural environments, there are several ways in which unusable or uninformative video data can be captured. Unusable video occurs when video is captured in an inadequately lit environment. In this scenario, even with the camera’s autoexposure set to maximum, the signal to noise ratio of the captured video is too low to extract any meaningful signal. Uninformative video occurs when the camera is not being worn. For example, at the beginning or end of a recording session, the user might put the camera down while it is still recording. Hence, we need to detect periods of no camera motion. We label, but preserve, unusable periods. This means absolute timestamps are preserved throughout the video while still enabling extracted data to be ignored during the unusable periods. To provide both a means to store these time segment labels but also to provide an intuitive visualization, we log unusable/uninformative periods in a subtitle file (SRT format) accompanying each video file. This means that an operator can playback the video and see informative labels for periods that will be excluded from analysis. Finally, it is possible to wear the adult camera upside down. When this accidentally occurs, we need to detect the video inversion and correct it before attempting to extract any information from the videos. We now describe how we automatically detect each of these cases.

**Dark period detection.** During search for the synchronization signal, we already compute mean intensity per frame and color channel. This is done efficiently using global average pooling on the GPU so has very low computational cost. We further average over color channels providing a scalar time series indicating the mean average brightness over the video. To detect dark periods, we simply threshold this signal and label frames where the mean brightness is below the threshold. The threshold value is chosen by manually selecting dark and light periods and finding the optimal separation between the two. In practice, we threshold on a value of T = 0.15 (with intensities normalized between 0 and 1).

**Static period detection.** A static camera can occur when the infant is sleeping, or when the HMC has been removed and left recording on a stationary surface. A static camera means that static scene elements do not move in the image, though dynamic scene contents may still move (for example people walking in front of the camera). To detect static scenes we compute dense optical flow between adjacent frames with a light version of the RAFT neural network [127], in which each frame is sampled once per second of the session recording. Scenes with a static camera are generally characterized by a lack of motion at the image boundary, where motion due to dynamic objects is minimal. Therefore, we extract only the image boundary of the optical flow (defined as a 5 pixel border around the image frame), which we split into 20 equally sized segments, to evaluate boundary motion. Subsequently, we compute the mean absolute value of the flow magnitude in these segments, and find the number of segments where this value is below a predetermined threshold value (which we set to 2 pixels), signifying a lack of motion. If the number of these segments is greater than or equal to 5 segments, then we classify the image pair as being *static*. We use a validation set of over 8k frames to achieve >98% classification accuracy.

**Inversion detection.** Due to user error, videos can be inverted due to participants wearing HMCs upside-down. Usually, entire videos are inverted though occasionally a user corrects the orientation part way through. It is vital to detect inverted videos since any subsequent processing for tasks such as face detection are likely to be more degraded since face detection networks are not usually trained on inverted faces. Our assumption is that inversion should be evident from scene contents visible in most frames (for example regions recognized as floor or objects on the floor should be below walls, people and ceiling). We use a convolutional neural network (CNN) to recognize such scene contents and their arrangement. The CNN is trained to take an image as input and to output a single scalar probability indicating the probability that the frame is inverted. For training, we manually identify a set of inverted and non-inverted videos captured in diverse and natural environments. From these videos, we form a dataset of 16k labeled frames, of which half are inverted and half are normal orientation. For our CNN architecture, we use the B0 variant of the EfficientNet model [128]. We use a model that was pre-trained for ImageNet classification such that we only need to fine-tune it for our task. We replace the final classification layer with a fully connected layer with a single output and sigmoid activation. We perform the fine-tuning using binary cross-entropy loss, the Adam optimizer, a batch size of 16 and downsample video frames to size 224 by 224 prior to input. We train for 5 epochs. We use an unseen validation set comprising an additional 4k frames (evenly balanced between inverted and non-inverted) on which we achieve >99% classification accuracy and >99% precision and recall. Within our pipeline, we use this model to classify one frame per second over a session as either inverted or normal orientation. We log the session as wholly inverted if it is greater than 80 percent inverted from this sampling. Sessions with a high, but <80%, predicted inverted frame rate are tagged for later verification by a human.

## 4. Discussion

Child development involves complex and interactive processes that occur over multiple time scales (e.g., [11,14,15,16]). These processes require a deeper understanding of how infants actively explore their environment. As infants develop their cognitive, social, language and motor capabilities, they interact with their environment, carers and other people from their unique (and developing) egocentric perspective. This interaction, in turn, dynamically changes the information that they attend to and acquire as they develop (for a review, see [7]). Although a strength of non-natural lab-based studies is tight experimental control, they fail to capture the wealth of data that can help researchers understand infants’ trajectory for critical developmental areas ranging from executive function to emotion processing and social learning. Thus, a *naturalistic approach*, in which large amounts of multimodal data are collected in the wild, is needed to advance developmental science. However, to date, there is no user-friendly and unobtrusive technology to densely and reliably sample life in the wild.

### 4.1. New Tools for Developmental Research

To address this gap, we developed the EgoActive platform which provides wireless and wearable sensors to measure infants’ and carers’ egocentric auditory and visual experiences concurrently with their ongoing cardiac activity and body movements. The head-mounted cameras to record egocentric perspectives were designed to be lightweight and small so that they can be used across a large age range from infants (as young as 5–6 months) to adults. Despite this important small-form factor for the naturalistic approach, the lens has a sufficient FOV to capture the visual environment, and the camera records high-resolution images at 30 Hz continuously for several hours. A similar design philosophy was taken for the body sensors to record ECG and acceleration across a large age range. We used a two-electrode configuration to ensure ease of use with infant-friendly electrodes while allowing for reliable ECG sampling at 250 Hz continuously for several hours. This sampling rate can accurately capture R peaks, and allow for more advanced HR variability analyses [104,105]. Acceleration is accurately recorded by a calibrated accelerometer at 65 Hz. The casing material for the HMCs and body sensors are lightweight and comfortable for users, particularly infants, yet durable enough to insulate and protect camera lens, circuit boards, batteries and internal cables. The EgoActive platform includes a portable base unit to synchronize different devices, and act as a charging station and data backup. Lastly, we developed open source software tools to facilitate critical preprocessing stages for the raw data, including temporally aligning data from different devices and data cleaning (see Appendix A for a list).

We validated the EgoActive platform (see Section 2.5) to ensure that our custom designed wearable sensors unobtrusively and reliably capture infants’, toddlers’ and adults’ egocentric perspectives and physiological states during daily activities (e.g., play or tea time). For the HMC, we showed that the camera lens’ FOV captured more than 80% of user fixations recorded by a head-mounted eye tracking system [106]. Although our EgoActive HMC only measures the scene as a function of head direction, the comparison with the eye-tracking data in our validation study suggests that the scenes captured by the HMC include the majority of fixations that users are likely to make. However, this is a limitation that should be considered for specific studies. For ECG and acceleration, we measured data concurrently from our EgoActive device and the Biosignalplux (Biosignalsplux, Portugal) device which has been used in previous studies with adults (e.g., [129,130,131]). Our device performed comparably to the Biosignalplux (Biosignalsplux, Portugal) device. We also used an ECG simulator to demonstrate that the EgoActive sensor had reliable recordings over a long period relative to a ground truth. Finally, we asked families to use the EgoActive platform for one week to gauge user experience. A majority of the caregivers reported that the platform was easy to use and operate, comfortable and did not interfere with their daily activities. The caregivers also reported that 6-month-old infants infrequently removed the devices. Thus, following our specification, other researchers can construct the EgoActive platform for use in their research without further need for validation.

### 4.2. Challenges for the EgoActive Platform

We designed the EgoActive platform to be well suited for developmental research ‘in the wild’. However, there remain challenges in using our platform for the naturalistic approach. First, despite its user-friendliness (see Section 2.5), our platform may still require that users are comfortable with technology and that they have some minimum level of technical competency. Here, we think that researchers can improve overall usability; for example, by familiarizing users with the platform, and providing training with the sensor devices (as we did). Second, there can be challenges with infants (and adults) interacting with the sensors beyond their intended use (e.g., older infants may remove the sensors). We provide software solutions to help detect these periods (see Section 3.2) and will continue to improve on these solutions. Third, there can be challenges with maintaining the different components. We designed the platform to use off-the-shelf components where possible, and otherwise readily available materials. The technology used to manufacture the components at a relatively small scale is also widely available. Lastly, there can be privacy concerns as the platform records video and audio from their everyday environment (e.g., home). Researchers should therefore have good ethical protocols in place in line with their institution recommendations and country legislation concerning data privacy and reporting issues.

### 4.3. Summary and Research Potential

To summarize, the EgoActive platform allows researchers to measure dynamic changes to individuals’ natural environment from their egocentric perspective synchronized to corresponding changes to their physiological states. The synchronized data from our platform can be used to tease apart the complex processes that are important for development. For example, changes to heart rate have been shown to relate to infants’ attentional states (e.g., [31,32,33,34]). The synchronized video and ECG data allow researchers to determine whether different stimuli in the environment (e.g., faces, toys or people) can predict heart-rate fluctuations and possibly these attentional states. Given that the platform is easy to use, infants can be tested longitudinally to quantify changes to their egocentric experiences of the environment as they develop more motor coordination. The capacity to record concurrently from infants and caregivers will allow researchers to study parent-child interactions with richer data collected in natural situations and with any unintended influences by researchers mitigated (e.g., [66,132,133]). 

The data collected in the wild can complement lab-based experiments, e.g., EEG studies that provide measures of neural development [134]. Lastly, as noted above, researchers can collect large and rich multimodal data using our platform. This wealth of data can be used to quantify statistical regularities in infants’, toddlers’ and children’s auditory and visual environment. The multimodal data can also contribute to improving the output of mathematical models and machine-learning systems that aim to automatically process auditory or visual input for specific tasks (e.g., detect facial emotions or detect certain sounds such as crying), particularly from infants’ egocentric perspective. Most existing training data are based on data scraped from the internet and may therefore not generalize to natural situations. Furthermore, little to none of these data contain information actively sampled by infants and children from their environment.

**Figure 25:**
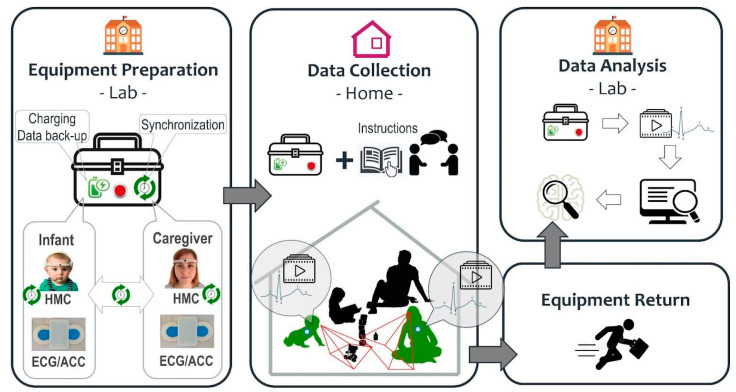
A flow diagram illustrating one possible deployment option of the EgoActive platform for research. In the research lab, the equipment is prepared, including the HMCs, body sensors, base unit, electrodes and instructional materials. The platform can be delivered to a home or picked up from the lab, and a researcher can instruct caregivers on its use. The caregiver and infant wear the HMC and sensor for several hours per day sampling life in the wild, using the base unit to synchronize all four devices per recording session and for data backup. The platform is then returned to the research lab. Software tools provided with the platform are used to temporally align the data from the devices, and then preprocess and clean the temporally aligned HMC, ECG and acceleration data for further analyses to address specific research questions.

We highlight that the EgoActive platform can be easily deployed, as illustrated in Figure 25. This important design feature means that researchers can also systematically test and compare populations from a wide range of cultural and socio-economical environments, beyond the WEIRD countries. Indeed, very little is known about the extent to which existing developmental models, based on lab data collected in WEIRD countries, explain the cognitive and socio-emotional development of children worldwide (e.g., [17,18,135]). These newly developed tools for the naturalistic approaches are crucial for achieving step changes in understanding child development across longer age spans through larger-scale studies.

## Figures and Tables

**Table 1:** Age distribution across the HMC with narrow or wide FOV. Values show the mean across participants in months and days.

	Narrow-FOV HMC		Wide-FOV HMC	
Age	*N*	Mean Age	*N*	Mean Age
		(Months—m, Days—d)		(Months—m, Days—d)
6 months	10	6 m, 8 d	NA	NA
12 months	8	12 m, 15 d	8	11 m, 28 d
24 months	10	24 m, 28 d	6	25 m, 2 d
36 months	5	37 m, 5 d	4	38 m, 2 d
Adults	9	306 m, 28 d	9	361 m, 9 d

**Table 2:** Age distribution. Values show the mean across participants in months and days.

Age	*N*	Mean Age
		Months—m, Days—d
3 months	6	3 m, 27 d
6 months	5	6 m, 41 d
9 months	3	8 m, 85 d
12 months	8	13 m, 0 d
24 months	3	25 m, 18 d
36 months	5	37 m, 71 d

**Figure A1:**
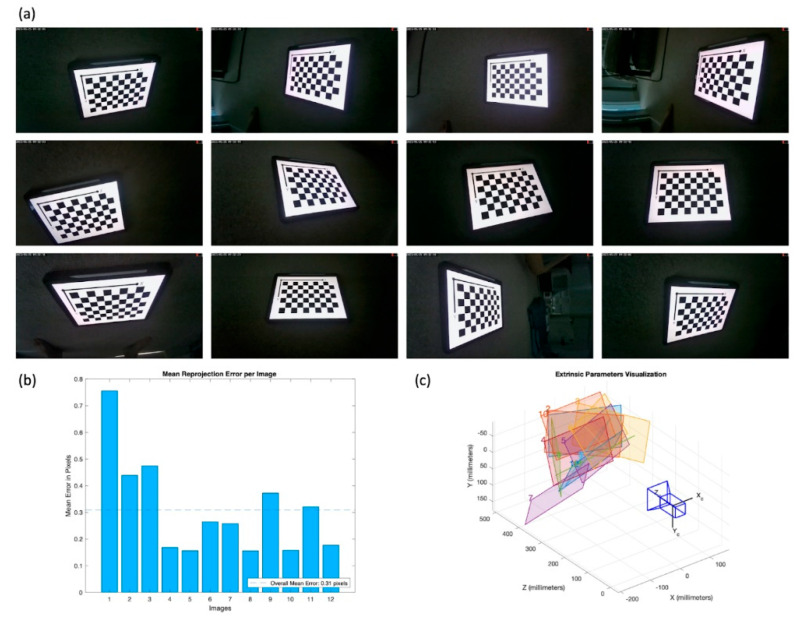
Camera calibration results for the wide-FOV lens: (**a**) captured calibration images, (**b**) mean reprojection error for each image, and (**c**) estimated positions of the calibrated target relative to the camera.

**Figure A2:**
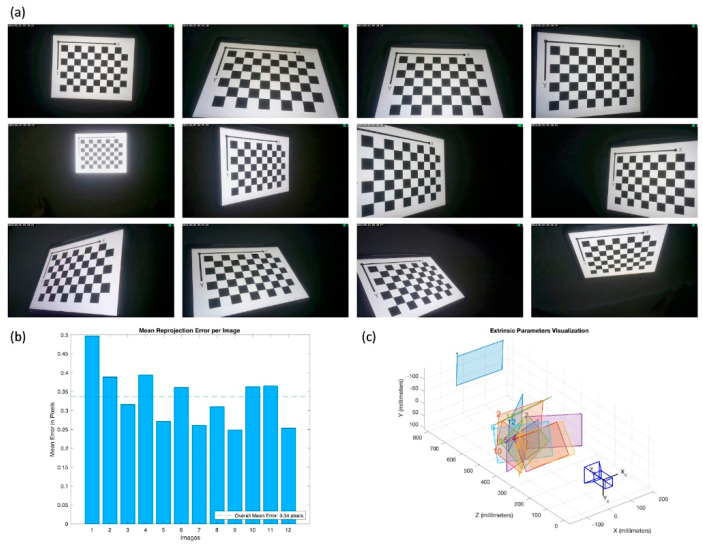
Camera calibration results for the narrow-FOV lens: (**a**) captured calibration images, (**b**) mean reprojection error for each image, and (**c**) estimated positions of the calibrated target relative to the camera.

**Figure A3:**
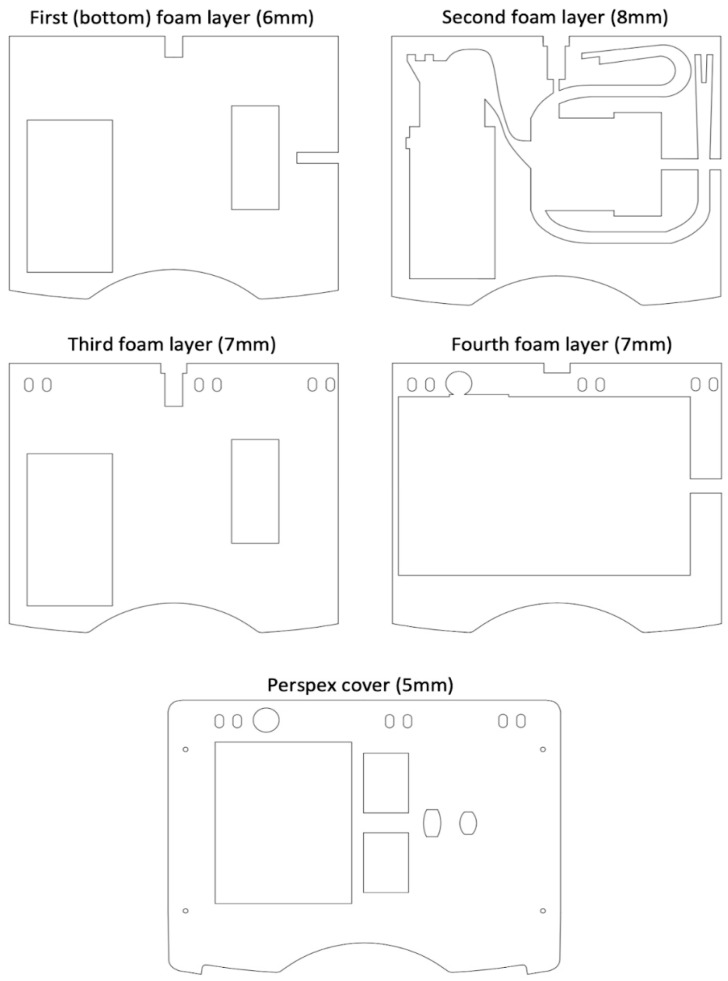
The components of base unit.

## Data Availability

All data, hardware design and software will be made available upon reasonable request sent to the corresponding author (E.G.).

## Appendix A

The following additional resources will be made available to researchers upon request:

1. A sample dataset comprising a raw video and body sensor recording and the resulting synchronized time series after applying our preprocessing pipeline.
2. Python source code for:
  (a) Video resampling, stitching and synchronization signal detection,
  (b) Detection of dark/static/inverted videos or video segments,
  (c) Conversion of raw .dat body sensor files into human-readable .txt files,
  (d) Extraction, processing, and quality assessment of heart rate from raw ECG signal, and
  (e) Extraction and processing of accelerometer files.
3. AutoCAD designs for base unit foam insert layers.
4. Java source code for Android tablet backup and synchronization app.
5. Body sensor manufacturing files:
  (a) KiCAD project files for the body sensor body sensor circuit board design.
  (b) Bill of Materials.
  (c) Arduino sketch, the program code which drives the body sensor processor.
  (d) Python script to read and convert the raw binary .dat files into human-readable .txt files for heart rate and accelerometer data.
  (e) CAD files for the body sensor case design (injection molding and 3D printing).
6. HMC manufacturing files:
  (a) System diagram,
  (b) Bill of Materials,
  (c) CAD designs for the HMC casings (injection molding and 3D printing), and
  (d) Instructions for assembling the HMC.

## Appendix B

### Camera Calibration Results

We performed a geometric calibration of both the wide- and narrow-FOV lenses using a pinhole perspective model with two radial and two tangential distortion parameters. For each lens, we moved the camera around a fixed calibration target to capture the target from different perspectives. This target was a planar black and white checkerboard pattern with 7 rows and 10 columns (35 black and 35 white squares). We then extracted 12 frames which captured the target from 12 different perspectives with the camera’s native resolution of 1920 × 1080 pixels. We show visual results in Figure A1 and Figure A2 for the wide- and narrow-FOV lens, respectively. In (a), we show the 12 captured images. In (b), we show the mean reprojection errors per image for the checkerboard corners. For both cameras, the calibration has subpixel level accuracy (mean error approximately 0.3 pixels). In (c), we show the estimated poses of the calibration target relative to the camera. This shows the range of poses used within the calibration.

The calibration provides the following estimates for the intrinsic parameters of the cameras:

**Table A1:** Camera calibration parameters for the wide-FOV and narrow-FOV lenses.

	Wide-FOV Lens	Narrow-FOV Lens
Focal length (x)	972.3 pixels	1571.5 pixels
Focal length (y)	971.7 pixels	1569.6 pixels
Principal point (x)	967.3 pixels	946.3 pixels
Principal point (y)	525.8 pixels	512.0 pixels
Radial distortion 1	0.0638	0.0462
Radial distortion 2	−0.0979	−0.0768
Tangential distortion 1	−3.3806 × 10^−4^	−5.42 × 10^−4^
Tangential distortion 2	−8.75 × 10^−5^	3.01 × 10^−4^

As expected, the wide FOV of the adult camera is reflected in the shorter focal length. The resulting increased fisheye distortion is reflected in the larger magnitude of the radial distortion coefficients.

## Appendix C

### Single Run through of the App

The experience of using the Android application varies depending on the roles of setup, user, and researcher. The setup process involves the initial configuration of the application on a new Android device.

**Table A2:** User actions within the Android application.

Role	Process	Actions
Administrators	Initial setup	Perform typical Android setup procedure Insert SD card Install application from Github Give application full administrator rights in settings
Administrators	Data collection preparations	Receive the box Retrieve cameras/body sensors from inside box Enter ‘Admin Room’ in application Initialize cameras Create save location on tablet Exit Admin Room Enter User Home
End users	Data collection in the natural environment	Receive box Receive short training by administrators Initialize the synchronization process to begin recording Record data Backup data Repeat until time to give box back

- Time/Counter verification

To select the GIF displayed on synchronization, the time, date and counter are encoded into binary as shown in Source code listing A1. This returns a 10 bit binary string that is used to select a synchronization GIF from the list.

**Source code listing A1.** Java code snippet for converting date, time and counter into 10 bit binary string.

- Timer/Delay (Discussing the several minute requirement through sync to stop overlapping files)

The application relies heavily on the public API SimpleStorage to work due to the nature of file transmission within Android, the file transfer speed can vary from device to device, and it takes an average of 30 s to transfer a 5 GB file; however, within some extreme circumstances during testing, it could take up to 15 min to transfer a file, depending on the efficiency of the hardware/how full the device was. For this reason, there is an in-built warning regarding the transfer of files and requiring up to 30 min. Within testing, should a new transfer be started before the previous finished, it would create overlapping folders which overall caused the application to run incredibly slowly and resulted in missing data.

## Appendix D

### CAD Designs for the Four Foam Insert Layers and Perspex Cover for the Base Unit Case

(See Section 2.4)

The base unit is housed within a WAG TEKNO 2007 polypropylene carry case (external dimensions: 340 mm × 275 mm × 83 mm; W.AG Funktion + Design GmbH, Germany). The bottom of the case contains the hardware components while the lid provides storage and carry space for four HMCs and two body sensors. To provide a rigid mounting surface, we screw and glue 24 mm × 28 mm wooden inserts along the two sides of the case with a chamfered edge to fit the curved internal corners of the case. The electronic components are held in place and cushioned by four layers of polyethylene closed cell foam which we laser cut according to custom designs (see below). These are glued to the case and the layer below, bringing the top of the final foam layer flush with the wooden inserts. Everything is held in place by a Perspex sheet which is screwed to the wooden inserts, providing cut outs for the tablet screen, charge and backup cables and access to the tablet power button. Importantly, the Perspex sheet also has cutouts specifically sized for the HMCs and sensors to ensure ease and reliability of synchronizing the devices (Figure 18 main text).

## References

[1] Jayaraman S., Smith L.B., Lockman J.J., Tamis-LeMonda C.S. (2020). The infant’s visual world: The everyday statistics for visual learning. The Cambridge Handbook of Infant Development: Brain, Behavior, and Cultural Context.

[2] Bronfenbrenner U. (1977). Toward an experimental ecology of human development. Am. Psychol..

[3] Bronfenbrenner U. (1986). Ecology of the family as a context for human development: Research perspectives. Dev. Psychol..

[4] Dahl A. (2017). Ecological commitments: Why developmental science needs naturalistic methods. Child Dev. Perspect..

[5] Oller D.K., Niyogi P., Gray S., Richards J.A., Gilkerson J., Xu D., Yapanel U., Warren S.F. (2010). Automated vocal analysis of naturalistic recordings from children with autism, language delay, and typical development. Proc. Natl. Acad. Sci. USA.

[6] Willems E.P. (1967). Toward an explicit rationale for naturalistic research methods. Hum. Dev..

[7] Smith L.B., Jayaraman S., Clerkin E., Yu C. (2018). The developing infant creates a curriculum for statistical learning. Trends Cogn. Sci..

[8] Cristia A. (2023). A systematic review suggests marked differences in the prevalence of infant-directed vocalization across groups of populations. Dev. Sci..

[9] Long B., Goodin S., Kachergis G., Marchman V.A., Radwan S., Sparks R., Xiang V., Zhuang C., Hsu O., Newman B. The BabyView Camera: Designing a New Head-Mounted Camera to Capture Children’s Early Social and Visual Environment. psyarxiv.com/238jk.

[10] Smith L.B., Yu C., Yoshida H., Fausey C.M. (2015). Contributions of head-mounted cameras to studying the visual environments of infants and young children. J. Cogn. Dev..

[11] Tamis-LeMonda C.S. (2023). The mountain stream of infant development. Infancy.

[12] Wass S.V., Goupil L. (2022). Studying the developing brain in real-world contexts: Moving from castles in the air to castles on the ground. Front. Integr. Neurosci..

[13] Maitha C., Goode J.C., Maulucci D.P., Lasassmeh S.M., Yu C., Smith L.B., Borjon J.I. (2020). An open-source, wireless vest for measuring autonomic function in infants. Behav. Res. Methods.

[14] Smith L.B., Thelen E. (2003). Development as a dynamic system. Trends Cogn. Sci..

[15] Thelen E., Port R.F., Van Gelder T. (1995). Time-scale dynamics and the development of an embodied cognition. Mind as Motion: Explorations in the Dynamics of Cognition.

[16] Thelen E., Corbetta D., Granott N., Parziale J. (2002). Microdevelopment and dynamic systems: Applications to infant motor development. Microdevelopment: Transition Processes in Development and Learning.

[17] Keller H. (2018). Parenting and socioemotional development in infancy and early childhood. Dev. Rev..

[18] Singh L., Cristia A., Karasik L.B., Rajendra S.J., Oakes L.M. (2023). Diversity and representation in infant research: Barriers and bridges toward a globalized science of infant development. Infancy.

[19] Geangu E., Wright J.D. (2015). Development of empathy during early childhood across cultures. International Encyclopedia of the Social and Behavioral Sciences.

[20] Gibbins I. (2013). Functional organization of autonomic neural pathways. Organogenesis.

[21] Sharkey K., Pittman Q. (1996). The autonomic nervous system: Peripheral and central integrative aspects. Comprehensive Human Physiology: From Cellular Mechanisms to Integration.

[22] Berntson G.G., Cacioppo J.T., Quigley K.S. (1993). Respiratory sinus arrhythmia: Autonomic origins, physiological mechanisms, and psychophysiological implications. Psychophysiology.

[23] Jennings R.J., van der Molen M.W. (2002). Cardiac timing and the central regulation of action. Psychol. Res..

[24] Kleinow J., Smith A. (2006). Potential interactions among linguistic, autonomic, and motor factors in speech. Dev. Psychobiol..

[25] Peters H.F., Hulstijn W. (1984). Stuttering and anxiety: The difference between stutterers and nonstutterers in verbal apprehension and physiologic arousal during the anticipation of speech and non-speech tasks. J. Fluency Disord..

[26] Gomez I.N., Flores J.G. (2020). Diverse patterns of autonomic nervous system response to sensory stimuli among children with autism. Curr. Dev. Dis. Rep..

[27] Heilman K.J., Harden E.R., Zageris D.M., Berry-Kravis E., Porges S.W. (2011). Autonomic regulation in fragile X syndrome. Dev. Psychobiol..

[28] Imeraj L., Antrop I., Roeyers H., Swanson J., Deschepper E., Bal S., Deboutte D. (2012). Time-of-day effects in arousal: Disrupted diurnal cortisol profiles in children with ADHD. J. Child Psychol. Psychiatry.

[29] Van Goozen S.H., Matthys W., Cohen-Kettenis P.T., Buitelaar J.K., Van Engeland H. (2000). Hypothalamic-pituitary-adrenal axis and autonomic nervous system activity in disruptive children and matched controls. J. Am. Acad. Child Adolesc. Psychiatry.

[30] Mulkey S.B., Dú Plessis A. (2018). The critical role of the central autonomic nervous system in fetal-neonatal transition. Semin. Pediatr. Neurol..

[31] Coles M.G. (1972). Cardiac and respiratory activity during visual search. J. Exp. Psychol..

[32] Porges S.W., Humphrey M.M. (1977). Cardiac and respiratory responses during visual search in nonretarded children and retarded adolescents. Am. J. Ment. Defic..

[33] Porges S.W., Raskin D.C. (1969). Respiratory and heart rate components of attention. J. Exp. Psychol..

[34] Richards J.E., Casey B.J. (1991). Heart rate variability during attention phases in young infants. Psychophysiology.

[35] Zantinge G., van Rijn S., Stockmann L., Swaab H. (2017). Psychophysiological responses to emotions of others in young children with autism spectrum disorders: Correlates of social functioning. Autism Res..

[36] Zantinge G., van Rijn S., Stockmann L., Swaab H. (2017). Physiological arousal and emotion regulation strategies in young children with autism spectrum disorders. J. Autism Dev. Disord..

[37] Berg W.K., Berg K.M., Osofsky J.D. (1987). Psychophysiological development in infancy: State, startle, and attention. Handbook of Infant Development.

[38] Graham F., Kimmel H., Olst E.H., Orlebeke J.F. (2021). Distinguishing among orienting, defense, and startle reflexes. The Orienting Reflex in Humans.

[39] Graham F., Anthony B., Zeigler B. (1983). Orienting and Habituation: Perspectives in Human Research. Psychol. Res..

[40] Porges S.W. (1976). Peripheral and neurochemical parallels of psychopathology: A psychophysiological model relating autonomic imbalance to hyperactivity, psychopathy, and autism. Adv. Child Dev. Behav..

[41] Porges S.W., Bohrer R.E., Cheung M.N., Drasgow F., McCabe P.M., Keren G. (1980). New time-series statistic for detecting rhythmic co-occurrence in the frequency domain: The weighted coherence and its application to psychophysiological research. Psychol. Bull..

[42] Von Bargen D.M. (1983). Infant heart rate: A review of research and methodology. Merrill-Palmer Q..

[43] Lansink J.M., Richards J.E. (1997). Heart rate and behavioral measures of attention in six-, nine-, and twelve-month-old infants during object exploration. Child Dev..

[44] Richards J.E., Gibson T.L. (1997). Extended visual fixation in young infants: Look distributions, heart rate changes, and attention. Child Dev..

[45] Richards J.E. (2003). Attention affects the recognition of briefly presented visual stimuli in infants: An ERP study. Dev. Sci..

[46] Xie W., Mallin B.M., Richards J.E. (2018). Development of infant sustained attention and its relation to EEG oscillations: An EEG and cortical source analysis study. Dev. Sci..

[47] Amso D., Scerif G. (2015). The attentive brain: Insights from developmental cognitive neuroscience. Nat. Rev. Neurosci..

[48] Oakes L.M., Tamis-Lemonda C.S.L., Jeffrey J. (2023). The development of visual attention in infancy: A cascade approach. Advances in Child Development and Behavior.

[49] Perry R.E., Braren S.H., Rincón-Cortés M., Brandes-Aitken A.N., Chopra D., Opendak M., Alberini C.M., Sullivan R.M., Blair C. (2019). Enhancing executive functions through social interactions: Causal evidence using a cross-species model. Front. Psychol..

[50] Posner M.I., Rothbart M.K. (2000). Developing mechanisms of self-regulation. Dev. Psychopathol..

[51] Reynolds G.D., Romano A.C. (2016). The development of attention systems and working memory in infancy. Front. Syst. Neurosci..

[52] Rose S.A., Feldman J.F., Jankowski J.J. (2001). Visual short-term memory in the first year of life: Capacity and recency effects. Dev. Psychol..

[53] Ruff H.A., Lawson K.R. (1990). Development of sustained, focused attention in young children during free play. Dev. Psychol..

[54] Smith L.B., Yu C. (2013). Visual attention is not enough: Individual differences in statistical word-referent learning in infants. Lang. Learn. Dev..

[55] Ursache A., Blair C., Stifter C., Voegtline K. (2013). Emotional reactivity and regulation in infancy interact to predict executive functioning in early childhood. Dev. Psychol..

[56] Brandes-Aitken A., Braren S., Swingler M., Voegtline K., Blair C. (2019). Sustained attention in infancy: A foundation for the development of multiple aspects of self-regulation for children in poverty. J. Exp. Child Psychol..

[57] Porges S.W. (2007). The polyvagal perspective. Biol. Psychol..

[58] Beauchaine T. (2001). Vagal tone, development, and Gray’s motivational theory: Toward an integrated model of autonomic nervous system functioning in psychopathology. Dev. Psychopathol..

[59] Holzman J.B., Bridgett D.J. (2017). Heart rate variability indices as bio-markers of top-down self-regulatory mechanisms: A meta-analytic review. Neurosci. Biobehav. Rev..

[60] Porges S.W. (1995). Orienting in a defensive world: Mammalian modifications of our evolutionary heritage. A polyvagal theory. Psychophysiology.

[61] Blandon A.Y., Calkins S.D., Keane S.P., O’Brien M. (2008). Individual differences in trajectories of emotion regulation processes: The effects of maternal depressive symptomatology and children’s physiological regulation. Dev. Psychol..

[62] Calkins S.D. (1997). Cardiac vagal tone indices of temperamental reactivity and behavioral regulation in young children. Dev. Psychobiol..

[63] Calkins S.D., Keane S.P. (2004). Cardiac vagal regulation across the preschool period: Stability, continuity, and implications for childhood adjustment. Dev. Psychobiol..

[64] El-Sheikh M. (2005). Stability of respiratory sinus arrhythmia in children and young adolescents: A longitudinal examination. Dev. Psychobiol..

[65] Fabes R.A., Eisenberg N., Karbon M., Troyer D., Switzer G. (1994). The relations of children’s emotion regulation to their vicarious emotional responses and comforting behaviors. Child Dev..

[66] Gottman J.M., Katz L.F. (2002). Children’s emotional reactions to stressful parent-child interactions: The link between emotion regulation and vagal tone. Marriage Fam. Rev..

[67] Hessler D.M., Fainsilber Katz L. (2007). Children’s emotion regulation: Self-report and physiological response to peer provocation. Dev. Psychol..

[68] Scarpa A., Haden S.C., Tanaka A. (2010). Being hot-tempered: Autonomic, emotional, and behavioral distinctions between childhood reactive and proactive aggression. Biol. Psychol..

[69] Patriquin M.A., Lorenzi J., Scarpa A., Bell M.A. (2014). Developmental trajectories of respiratory sinus arrhythmia: Associations with social responsiveness. Dev. Psychobiol..

[70] Richter M., Lickenbrock D.M. (2021). Cardiac physiological regulation across early infancy: The roles of infant surgency and parental involvement with mothers and fathers. Infant Behav. Dev..

[71] Feldman R., Rosenthal Z., Eidelman A.I. (2014). Maternal-preterm skin-to-skin contact enhances child physiologic organization and cognitive control across the first 10 years of life. Biol. Psychiatry.

[72] Wagner N., Mills-Koonce R., Willoughby M., Propper C., Rehder P., Gueron-Sela N. (2017). Respiratory sinus arrhythmia and heart period in infancy as correlates of later oppositional defiant and callous-unemotional behaviors. Int. J. Behav. Dev..

[73] Aslin R.N. (2009). How infants view natural scenes gathered from a head-mounted camera. Optom. Vis. Sci..

[74] Braddick O., Atkinson J. (2011). Development of human visual function. Vision Res..

[75] Geisler W.S. (2008). Visual perception and the statistical properties of natural scenes. Annu. Rev. Psychol..

[76] Simoncelli E.P. (2003). Vision and the statistics of the visual environment. Curr. Opin. Neurobiol..

[77] Sinha P., Balas B., Ostrovsky Y. (2007). Discovering faces in infancy. J. Vis..

[78] Jayaraman S., Fausey C.M., Smith L.B. (2017). Why are faces denser in the visual experiences of younger than older infants?. Dev. Psychol..

[79] Jayaraman S., Smith L.B. (2019). Faces in early visual environments are persistent not just frequent. Vision Res..

[80] Johnson M.H. (2011). Interactive specialization: A domain-general framework for human functional brain development?. Dev. Cogn. Neurosci..

[81] Thelen E., Smith L.B., Damon W., Lerner R.M., Lerner R.M. (2007). Dynamic systems theories. Handbook of Child Psychology.

[82] Bigelow A.E., Rochat P. (2006). Two-month-old infants’ sensitivity to social contingency in mother–infant and stranger–infant interaction. Infancy.

[83] Werker J.F., Pons F., Dietrich C., Kajikawa S., Fais L., Amano S. (2007). Infant-directed speech supports phonetic category learning in English and Japanese. Cognition.

[84] Kretch K.S., Franchak J.M., Adolph K.E. (2014). Crawling and walking infants see the world differently. Child Dev..

[85] Campos J.J., Thein S., Owen D. (2003). A Darwinian legacy to understanding human infancy: Emotional expressions as behavior regulators. Ann. N. Y. Acad. Sci..

[86] Burbank B., McGregor D., Wild M. (2018). ‘My special, my special thing, and my camera!’ Using GoPro™ as a complementary research tool to investigate young children’s museum experiences. Mus. Soc..

[87] Ohnishi A., Murao K., Terada T., Tsukamoto M. (2019). A method for structuring meeting logs using wearable sensors. Internet Things.

[88] Prieto-Avalos G., Cruz-Ramos N.A., Alor-Hernández G., Sánchez-Cervantes J.L., Rodríguez-Mazahua L., Guarneros-Nolasco L.R. (2022). Wearable devices for physical monitoring of heart: A review. Biosensors.

[89] Kamble P.M. Life Logging: A Practicable Approach. Proceedings of the 2018 Fourth International Conference on Computing Communication Control and Automation.

[90] Rodin I., Furnari A., Mavroeidis D., Farinella G.M. (2021). Predicting the future from first person (egocentric) vision: A survey. Comput. Vis. Image Und..

[91] Yoon H., Kim S.K., Lee Y., Choi J. (2021). Google glass-supported cooperative training for health professionals: A case study based on using remote desktop virtual support. J. Multidiscip. Healthc..

[92] Borjon J.I., Schroer S.E., Bambach S., Slone L.K., Abney D.H., Crandall D.J., Smith L.B. (2018). A view of their own: Capturing the egocentric view of infants and toddlers with head-mounted cameras. J. Vis. Exp..

[93] Sullivan J., Mei M., Perfors A., Wojcik E., Frank M.C. (2022). SAYCam: A large, longitudinal audiovisual dataset recorded from the infant’s perspective. Open Mind.

[94] Kliper-Gross O., Gurovich Y., Hassner T., Wolf L. Motion interchange patterns for action recognition in unconstrained videos. Proceedings of the Computer Vision–ECCV 2012: 12th European Conference on Computer Vision.

[95] Ye V., Pavlakos G., Malik J., Kanazawa A. Decoupling human and camera motion from videos in the wild. Proceedings of the IEEE/CVF Conference on Computer Vision and Pattern Recognition.

[96] Grooby E., Sitaula C., Chang Kwok T.n., Sharkey D., Marzbanrad F., Malhotra A. (2023). Artificial intelligence-driven wearable technologies for neonatal cardiorespiratory monitoring: Part 1 wearable technology. Pediatr. Res..

[97] Celka P., Granqvist N., Schwabl H., Lutz M., Carton E., Baut J. Estimation of SpO2 at the Upper Arm. https://www.researchgate.net/publication/347751990_Estimation_of_SpO2_at_the_Upper_Arm.

[98] Geangu E., Hauf P., Bhardwaj R., Bentz W. (2011). Infant pupil diameter changes in response to others’ positive and negative emotions. PLoS ONE.

[99] Geangu E., Vuong Q.C. (2023). Seven-months-old infants show increased arousal to static emotion body expressions: Evidence from pupil dilation. Infancy.

[100] Crespo-Llado M.M., Vanderwert R.E., Geangu E. (2018). Individual differences in infants’ neural responses to their peers’ cry and laughter. Biol. Psychol..

[101] Hoehl S., Wahl S. (2012). Recording infant ERP data for cognitive research. Dev. Neuropsychol..

[102] Quadrelli E., Geangu E., Turati C. (2019). Human action sounds elicit sensorimotor activation early in life. Cortex.

[103] Geangu E., Senna I., Croci E., Turati C. (2015). The effect of biomechanical properties of motion on infants’ perception of goal-directed grasping actions. J. Exp. Child Psychol..

[104] (1996). Task force of the European society of cardiology and the north American society of pacing and electrophysiology. Heart rate variability. Standards of measurement, physiological interpretation, and clinical use. Eur. Heart J..

[105] Kwon O., Jeong J., Kim H.B., Kwon I.H., Park S.Y., Kim J.E., Choi Y. (2018). Electrocardiogram sampling frequency range acceptable for heart rate variability analysis. Healthc. Inform. Res..

[106] Franchak J.M., Kretch K.S., Soska K.C., Adolph K.E. (2011). Head-mounted eye tracking: A new method to describe infant looking. Child Dev..

[107] Holmqvist K., Örbom S.L., Hooge I.T., Niehorster D.C., Alexander R.G., Andersson R., Benjamins J.S., Blignaut P., Brouwer A.-M., Chuang L.L. (2023). Eye tracking: Empirical foundations for a minimal reporting guideline. Behav. Res. Methods.

[108] Jeyhani V., Mäntysalo M., Noponen K., Seppänen T., Vehkaoja A. Effect of different ECG leads on estimated R–R intervals and heart rate variability parameters. Proceedings of the 41st Annual International Conference of the IEEE Engineering in Medicine and Biology Society.

[109] Nelson B.W., Allen N.B. (2019). Accuracy of consumer wearable heart rate measurement during an ecologically valid 24-hour period: Intraindividual validation study. JMIR Mhealth Uhealth.

[110] Xu M., Sun W., Alam M. Security enhancement of secure USB debugging in Android system. Proceedings of the 2015 12th Annual IEEE Consumer Communications and Networking Conference.

[111] Wrótniak K. Android-gif-Drawable, GitHub Repository. https://github.com/koral--/android-gif-drawable.

[112] Hardiannico A. SimpleStorage, GitHub Repository. https://github.com/anggrayudi/SimpleStorage.

[113] Tomar S. (2006). Converting video formats with FFmpeg. Linux J..

[114] Pan J., Tompkins W. (1985). A real-time QRS detection algorithm. IEEE Trans. Biomed. Eng..

[115] Velayudhan A., Peter S. (2016). Noise analysis and different denoising techniques of ECG signal-a survey. J. Electron. Commun. Eng..

[116] Makowski D., Pham T., Lau Z.J., Brammer J.C., Lespinasse F., Pham H., Schölzel C., Chen S.A. (2021). NeuroKit2: A Python toolbox for neurophysiological signal processing. Behav. Res. Methods.

[117] Campero Jurado I., Lorato I., Morales J., Fruytier L., Stuart S., Panditha P., Janssen D.M., Rossetti N., Uzunbajakava N., Serban I.B. (2023). Signal quality analysis for long-term ECG monitoring using a health patch in cardiac patients. Sensors.

[118] Venkatachalam K., Herbrandson J.E., Asirvatham S.J. (2011). Signals and signal processing for the electrophysiologist: Part I: Electrogram acquisition. Circ. Arrhythmia Elect..

[119] Charlton P.H., Bonnici T., Tarassenko L., Clifton D.A., Beale R., Watkinson P.J. (2016). An assessment of algorithms to estimate respiratory rate from the electrocardiogram and photoplethysmogram. Physiol. Meas..

[120] Hirokawa J., Hitosugi T., Miki Y., Tsukamoto M., Yamasaki F., Kawakubo Y., Yokoyama T. (2022). The influence of electrocardiogram (ECG) filters on the heights of R and T waves in children. Sci. Rep..

[121] Van Gent P., Farah H., Van Nes N., Van Arem B. (2019). HeartPy: A novel heart rate algorithm for the analysis of noisy signals. Transp. Res. F—Traffic Psych. Behav..

[122] Rodrigues T., Samoutphonh S., Silva H., Fred A. A Low-Complexity R-peak Detection Algorithm with Adaptive Thresholding for Wearable Devices. Proceedings of the 25th International Conference on Pattern Recognition.

[123] Zhang Z., Li Z., Li Z. (2020). An improved real-time R-wave detection efficient algorithm in exercise ECG signal analysis. J. Healthc. Eng..

[124] Kramer L., Menon C., Elgendi M. (2022). ECGAssess: A Python-based toolbox to assess ECG lead signal quality. Front. Digit. Health.

[125] Rodrigues J., Belo D., Gamboa H. (2017). Noise detection on ECG based on agglomerative clustering of morphological features. Comput. Biol. Med..

[126] Jiang C., de Armendi J.T., Smith B.A. (2016). The immediate effect of positioning devices on infant leg movement characteristics. Pediatr. Phys. Ther..

[127] Teed Z., Deng J. Raft: Recurrent all-pairs field transforms for optical flow. Proceedings of the Computer Vision–ECCV 2020: 16th European Conference.

[128] Tan M., Le Q. Efficientnet: Rethinking model scaling for convolutional neural networks. Proceedings of the International Conference on Machine Learning.

[129] Gamboa H., Silva H., Fred A. (2014). HiMotion: A new research resource for the study of behavior, cognition, and emotion. Multimed. Tools Appl..

[130] Gamboa P., Varandas R., Rodrigues J., Cepeda C., Quaresma C., Gamboa H. (2022). Attention classification based on biosignals during standard cognitive tasks for occupational domains. Computers.

[131] Osório D.N., Viana-Soares R., Marto J.P., Mendonça M.D., Silva H.P., Quaresma C., Viana-Baptista M., Gamboa H., Vieira H.L. (2019). Autonomic nervous system response to remote ischemic conditioning: Heart rate variability assessment. BMC Cardiovasc. Disord..

[132] Atilla F., Alimardani M., Kawamoto T., Hiraki K. (2023). Mother-child inter-brain synchrony during a mutual visual search task: A study of feedback valence and role. Soc. Neurosci..

[133] Jeon H.-J., Peterson C.A., DeCoster J. (2013). Parent–child interaction, task-oriented regulation, and cognitive development in toddlers facing developmental risks. J. Appl. Dev. Psychol..

[134] Saby J.N., Marshall P.J. (2012). The utility of EEG band power analysis in the study of infancy and early childhood. Dev. Neuropsychol..

[135] Vuong Q.C., Geangu E. (2023). The development of emotion processing of body expressions from infancy to early childhood: A meta-analysis. Front. Cogn..

